# Large extracellular vesicle (EV) and neutrophil extracellular trap (NET) interaction captured in vivo during systemic inflammation

**DOI:** 10.1038/s41598-024-55081-x

**Published:** 2024-02-26

**Authors:** Weronika Ortmann, Anna Such, Iwona Cichon, Monika Baj-Krzyworzeka, Kazimierz Weglarczyk, Elzbieta Kolaczkowska

**Affiliations:** 1 Department of Experimental Hematology, Institute of Zoology and Biomedical Research, Jagiellonian University, Gronostajowa 9 Str, 30-387 Krakow, Poland; 2 Doctoral School of Exact and Natural Sciences, Jagiellonian University, Krakow, Poland; 3 Department of Clinical Immunology, Jagiellonian University Medical College, Wielicka 265 Str, 30-663 Krakow, Poland

**Keywords:** Innate immunity, Imaging the immune system, Neutrophils, Sepsis, Kupffer cells

## Abstract

Extracellular vesicles (EVs) and neutrophil extracellular traps (NETs) are pivotal bioactive structures involved in various processes including inflammation. Herein we report the interactions between EVs and NETs during murine endotoxemia studied in situ directly in the vasculature (cremaster muscle, liver sinusoids) using intravital microscopy (IVM). We captured NETs and EV release in real time by both non- and polarized neutrophils in liver but not in cremaster vasculature. When comparing numbers of circulating EVs of various origin (nanoparticle tracking analysis—NTA, flow cytometry) with those interacting with endothelium and NETs (IVM) we observed that whereas platelet and monocyte/macrophage-derived EVs dominate in blood and peritoneal lavage, respectively, mostly neutrophil-derived EVs interact with the vascular lining, NETs and leukocytes. Despite the interaction, NETs do not affect EV formation as NET release inhibition did not alter EV release. However, EVs inhibit NETs formation and in particular, erythrocyte-derived EVs downregulate NET release and this effect is mediated via Siglec-E-dependent interactions with neutrophils. Overall, we report that EVs are present in NETs in vivo and they do modulate their release but the process in not bidirectional. Moreover, EVs isolated from body fluids might not reflect their importance in direct endothelial- and leukocyte-related interactions.

## Introduction

Extracellular vesicles (EVs) are biologically active microstructures that are involved in numerous biological processes^1–3^. Extracellular vesicles are commonly subdivided into three main types: large EVs (0.1–2 μm in size), small EVs (30–150 nm) and apoptotic bodies. This is based on their heterogeneity, manner of formation, and content^3^. EVs are detected in numerous body fluids, not only the place of origin, due to their dynamic horizontal movement^3^. The latter is connected to their main function, i.e., the transport of bioactive cargo (proteins, genetic material) over long distances to target cells by which they are taken up^3,4^. Smaller EVs form multivesicular bodies during exocytosis while larger ones are secreted by direct budding from the cell membrane therefore markers specific to the releasing cell are present on their surface^5,6^. Moreover, migrasomes and elongated neutrophil-derived structures (ENDS) were described. Migrasomes are up to 3 µm in size and form at the ends or branches of the retraction fibers of polarized cells^7^. ENDS are formed from microvilli of nonpolarized rolling neutrophils^8^. Furthermore, the process of cell migration is associated with the formation of elongated uropods—trails^9,10^, including neutrophil-derived trails (NDTRs)^10,11^. Migrating neutrophils interact with endothelial cells through adhesion molecules and elongated uropods are detached from neutrophils, leaving chemokine-enriched EVs^10,11^.

Due to the wide spectrum of bioactive cargo carried, EVs are implicated in cell–cell communication maintaining homeostasis but also during inflammation^12^. In fact, during systemic inflammation, EVs are considered as disease mediators and prognostic markers^13^. Sepsis still presents a serious worldwide health problem^14^ and endotoxemia is its subtype induced by lipopolysaccharide (LPS) of Gram-negative bacteria accompanied by septic shock^15^.

A connection between EVs and sepsis was first shown over two decades ago in an endotoxemia pig model^16,17^. Since then, the topic was intensively explored due to EVs diversity, wide spectrum of activities and observation that they act as a double-edged sword being pro- and anti-inflammatory/pro-coagulant^18–20^. Similar characteristics are attributed to neutrophil extracellular traps (NETs) ejected from neutrophils to prevent the spread of pathogens by catching and immobilizing them. However, as inflammation progresses, NETs cause bystander damage and tissue/organ injury^21^. Decondensed DNA (extracellular DNA–exDNA) forms a backbone of NETs decorated with numerous antibacterial proteins/enzymes^22,23^. Two of them are also pivotal for NET formation i.e., peptidylarginine deiminase 4 (PAD4) and neutrophil elastase (NE). The PAD4 catalyzes the citrullination of histones whereas NE cleaves histones overall contributing to chromatin relaxation and decondensation^24,25^.

Interactions between EVs and NETs have been observed previously in isolated neutrophils. Wang et al.^26^ demonstrated that neutrophil-derived EVs can bind to NETs via histone-phosphatidylserine interplay^26^. EVs can also stimulate neutrophils to eject NETs ex vivo^27,28^. Furthermore, they can also be coated with histones communicating to other cells that infection is present^29^.

Herein we aimed at studying the interplay between EVs and NETs in situ where they are formed i.e., in vasculature of live mice with intravital microscopy (IVM). In particular large EVs were visualized. When describing our findings, unless otherwise stated, we refer to large extracellular vesicles as EVs. Whereas EVs were detectable in cremaster blood vessels, both EVs and NETs were present only in liver sinusoids. Therein we captured EVs release in real time during homeostasis and endotoxemia, and during the latter also NETs were released. These EVs correspond to structures described previously as migrasomes in the case of neutrophils. In vivo the majority of detected EVs originated from neutrophils (Ly6G^+^) and only some from macrophages (F4/80^+^) but hardly any platelet-derived EVs were observed interacting with the vasculature. This is in contrast to EVs detected in blood and peritoneal lavage. In blood CD41^+^ platelet EVs dominated and in the peritoneal lavage F4/80^+^ vesicles were the most numerous. In vivo we captured significant EV deposition in NETs, also in real time. Inhibition of NET formation did not alter EV release whereas injected exogenous EVs were mostly taken up by Kupffer cells (KCs) yet some were captured by NETs. Therefore, EVs impact on NETs was then studied ex vivo. EVs released by red blood cells (RBCs), but not other cells, inhibited NETs via Siglec-E-dependent interactions.

Overall, the study shows that numbers of circulating EVs differ from those interacting with endothelium and NETs in vivo in terms of their origin, and that the interplay between the two is not bidirectional with only EVs impacting NETs and not vice versa.

## Results

### Extracellular vesicles are present in blood plasma and peritoneal lavage during endotoxemia

**Figure 1:**
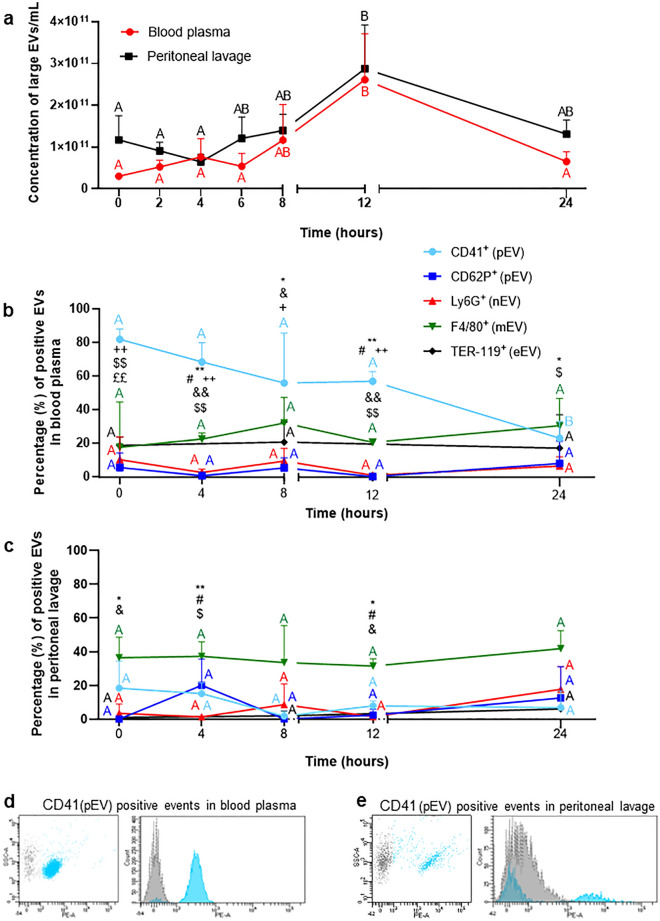
Quantification of the concentration of extracellular vesicles (EVs) and identification of their cellular markers during homeostasis and lipopolysaccharide (LPS)-induced systemic inflammation (*i.p.* 1 mg/kg b.w.) in C57BL/6 J mice. (**a**) Concentration of large EVs was estimated ex vivo with nanoparticle tracking analysis (NTA) in samples collected from blood plasma and peritoneal lavage. Percentage of EVs from (**b**) blood plasma and (**c**) peritoneal lavage positive for a given marker. Cellular markers tested: all platelets—CD41^+^ (light blue line; pEV); activated platelets—CD62^+^ (dark blue line; pEV); neutrophils—Ly6G^+^ (red line; nEV); monocytes/macrophages—F4/80^+^ (green line; mEV) and erythrocytes—TER-119^+^ (black line; eEV). (**d**,**e**) Exemplary dot-plots and histograms of fluorescently-labeled EVs (preceded by the FSC vs. SSC gating) positive for CD41^+^ (platelets) along with a corresponding isotype control (grey) obtained from mice with LPS-induced endotoxemia (8 h). Content of CD41^+^ EVs in (**d**) blood plasma and (**e**) peritoneal lavage. The results are expressed as the mean values ± SD. Values significantly different (*p˂0.05*) according to Kruskal–Wallis's test followed by Dunn's test (panel a for peritoneal lavage) or one-way ANOVA with Bonferroni multiple comparisons post hoc test (panel a for blood plasma and panels b,c) are designated by letters (different letters indicate statistical differences). Symbols (*, #, &, $, £, +) indicate statistically significant differences between the studied groups in the following order *Ly6G vs. F4/80; ^#^CD41 vs. F4/ 80; ^&^CD62P vs. F4/80; ^$^Ly6G vs. CD41; + CD41 vs. CD62P; ^£^CD41 vs. TER-119. The above differences are according to unpaired two-tailed Student’s t-test (*p ≤ *0.05*, **p ≤ *0.01*); n = 3–5. SSC-A—side scatter; PE-A—phycoerythrin.

**Figure 2:**
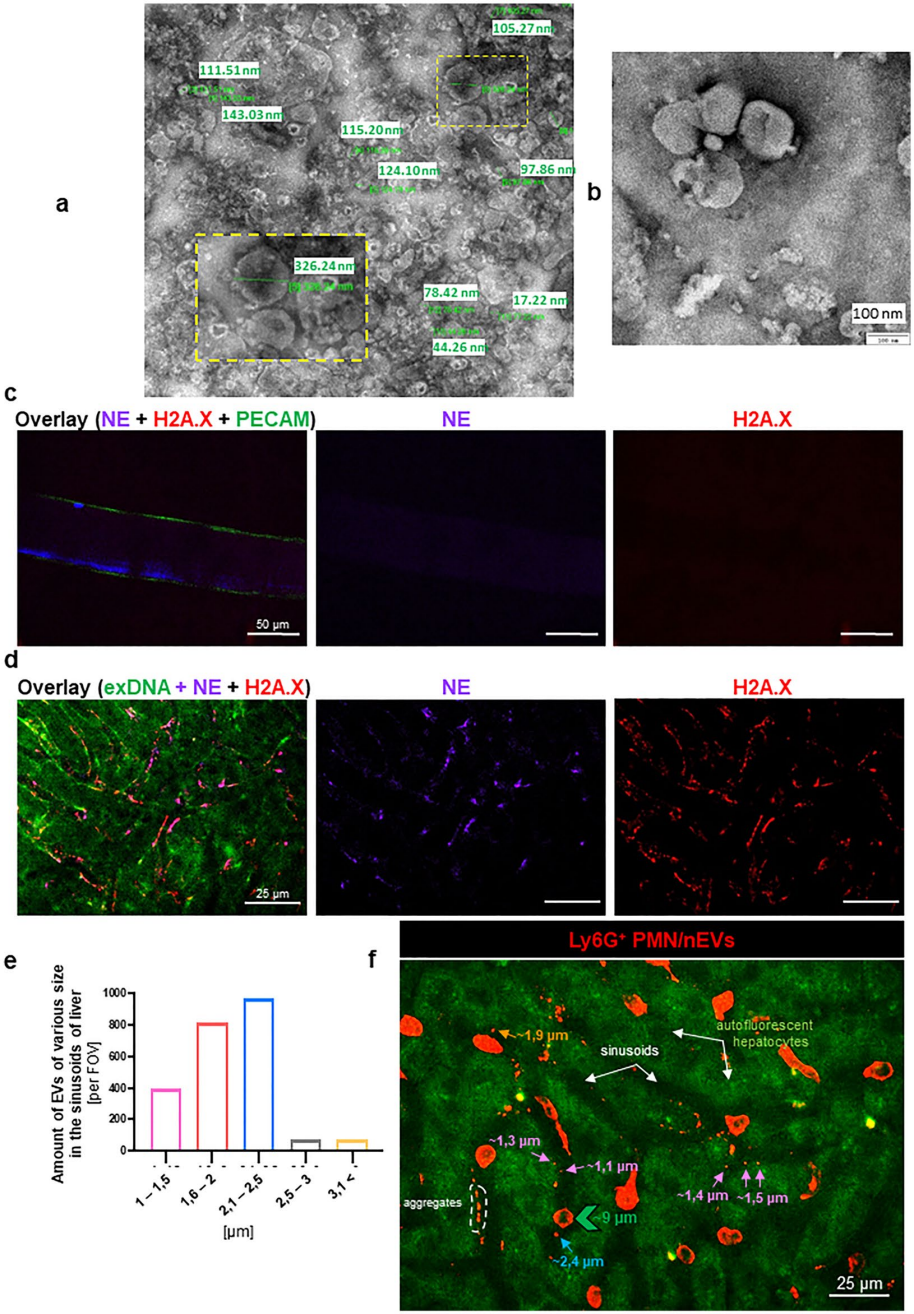
Morphology and size distribution of extracellular vesicles (EVs) in peritoneal lavage, and neutrophil extracellular trap (NET) formation in various vascular beds—in liver sinusoids and in the vessels of cremaster muscle. Exemplary images of EVs isolated from peritoneal lavage of C57BL/6 J mice with 8-h lipopolysaccharide (LPS)-induced endotoxemia and visualized with TEM: (**a**) exemplary size distribution in wide-field, and (**b**) a close-up; scale 100 nm. (**c**–**f**) C57BL/6 J healthy mice and mice with endotoxemia (*i.p.* 1 mg/kg b.w., 6 h) were subjected to in vivo imaging with Spinning Disk Confocal Intravital Microscopy (SD-IVM). Neutrophil extracellular traps in (**c**) vasculature of cremaster muscle and (**d**) liver sinusoids were identified by their main components, i.e., extracellular DNA (exDNA; green), neutrophil elastase (NE; violet), and histones (H2A.X; red). (**e**) Size distribution of all EVs in liver blood vessels (2000 vesicles from 21 mice were analyzed), and (**f**) exemplary image of liver sinusoids showing size range of individual neutrophil-derived EVs (Ly6G^+^ nEV; arrows; different colors were applied for various sizes) and nEV aggregates (white dotted line) in comparison to the neutrophil size (PMN; green arrowhead) (analyzed with ImageJ v1.53a software). The photos were taken under optical magnification of 20× (panels (**c**,**d**)) and 40× (panel (**f**)). The scale bar indicates 25 and 50 μm.

The number as well as size distribution of EVs (nanoparticle tracking analysis, NTA) isolated from blood plasma and peritoneal lavage changed over time but small ones (Supplementary Fig. S1) as well as large EVs (Fig. 1) were present both in control/healthy (time 0 h) and endotoxemic mice (Supplementary Fig. S2). In plasma of endotoxemic mice, an increase in large EVs was observed peaking at 12 h and then decreased to the levels detected in time 0 h (Fig. 1a). In uninflamed peritoneum, there was a tendency to a higher number of large EVs than in blood plasma, and upon induction of endotoxemia to their decline, but at 6–8 h of inflammation their numbers started to slowly increase reaching maximum at 12 h of inflammation (Fig. 1a). It might be peritoneal lavage contains also EVs released by abdominal organs whereas EVs are only one group of molecules present in blood^30^. Morphology and size of EVs in each body fluid was confirmed with transmission electron microscopy (TEM). EVs were of different sizes and shapes, mostly spheroid (Fig. 2a,b and Supplementary Fig. S4). Images obtained from peritoneum also show higher density of EVs in line with extracellular vesicles being with only a small fraction of all particles present in blood^30^.

NTA detected also the presence of the smallest vesicle population–small EVs, both during homeostasis and systemic inflammation (Supplementary Fig. S1). In plasma, their number fluctuated without correlation with inflammation progression as in the case of large EVs (Fig. 1a vs. Supplementary Fig. S1). Healthy mice (0 h) tended to have higher numbers of small EVs in blood plasma than in the peritoneal lavage (Supplementary Fig. S1). In the latter, small EVs showed better correlation with endotoxemia progression also reaching maximal counts at 12 h of inflammation but fluctuations were also apparent (Supplementary Fig. S1).

### Distribution of EVs of various origin in blood plasma and peritoneal lavage

Using flow cytometry, the cellular origin of large EVs present in blood plasma and peritoneal lavage was estimated (Fig. 1; controls Supplementary Fig. S3). EVs of the following cell origin were found in both body fluids: neutrophil (Ly6G^+^; nEV), monocyte/macrophage (F4/80^+^; mEV), erythrocyte (TER-119^+^; eEV) and platelet (CD41^+^ and CD62P^+^; pEV) (Fig. 1b–e). Whereas CD41 is expressed on all platelets, CD62P is present only on the activated ones^31,32^. In blood plasma CD41^+^ pEVs dominated over other EVs in the order all pEVs (CD41^+^) > mEVs > eEVs > nEVs > activated platelets CD62P^+^ EVs (Fig. 1b,d). Endotoxemia-related changes were only detected in CD41^+^ EV levels (a drop at 24 h) (Fig. 1b). In the peritoneal lavage mEVs dominated over other EVs in the following order—mEVs > all pEVs > activated pEVs > nEVs whereas eEVs were hardly detectable (Fig. 1c,e). Although the values did not reach statistical significance, more changes (tendencies) were observed in the case of peritoneal EVs. In particular, EVs originating from activated platelets displayed a tendency to be released particularly during first 4 h of endotoxemia whereas later on number of nEVs somewhat increased (Fig. 1c).

### Cremaster muscle: presence of extracellular vesicles, neutrophils and platelets during endotoxemia

In vasculature of cremaster muscle of healthy (0 h) mice, nEVs were not observed unlike in endotoxemic mice, both at 8 h (peak) and 12 h of inflammation (Supplementary Fig. S5a vs. b–d; Supplementary Video S1). We did not detect NETs in cremaster (Supplementary Fig. S5b,biii). In animals with endotoxemia, numerous nEVs adhered to the endothelium or were slowly moving (EV rolling) along the wall of the blood vessel (Supplementary Fig. S5b,bi; Supplementary Video S2) but we did not detect EV release in real time. Numerous interactions between neutrophils (Supplementary Fig. S5b,bi) and platelets (Supplementary Fig. S5b,bii) were also observed. Quantitative data concerning nEVs in vessels of cremaster muscle of healthy (0 h) and endotoxemic mice are presented in Supplementary Fig. S5d.

### Liver sinusoids: neutrophils, neutrophil-derived extracellular vesicles (nEVs) and NETs

**Figure 3:**
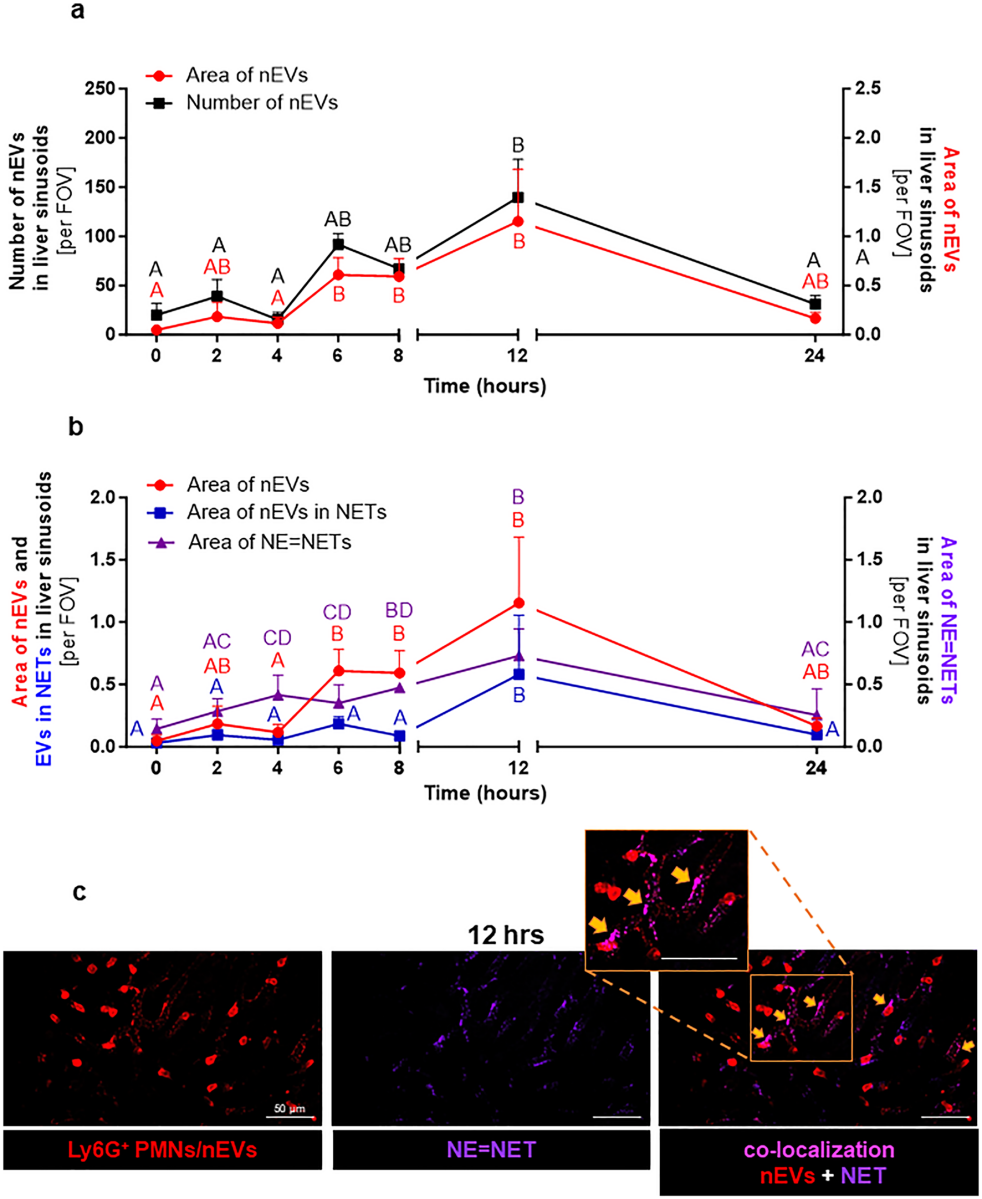
Quantification of the number/area of neutrophil-derived extracellular vesicles (nEVs) and neutrophil extracellular traps (NETs) during homeostasis and lipopolysaccharide (LPS)-induced systemic inflammation (*i.p.* 1 mg/kg b.w.) in C57BL/6 J mice. (**a**) Number or area (%) of nEVs and (**b**) area (%) covered by neutrophil elastase (NE = NETs) and nEVs present in NETs (co-localization) were in vivo acquired with Spinning Disk Confocal Intravital Microscopy (SD-IVM) directly in vasculature (sinusoids) of mice liver and analyzed with ImageJ v1.53a software. The results are expressed as the mean values ± SD. Values significantly different (*p˂0.05*) according to Kruskal–Wallis's test followed by Dunn's test (panel a for area (%) of nEVs and panel b) or one-way ANOVA with Bonferroni multiple comparisons post hoc test (panel a for number of nEVs) are designated by letters (different letters indicate statistical differences); n = 3. FOV—field of view. (**c**) Representative images of nEVs and NETs in liver sinusoids of endotoxemic mice (at 12 h). Single channels depicting nEVs (red structures) and neutrophils (red cells), neutrophil elastase = NET (violet) and nEV + NET co-localization (pink; exemplary areas of co-localization are marked with yellow arrows). The photos were taken under optical magnification of 40x. The scale bar indicates 20, 30, 40 or 50 μm.

**Figure 4:**
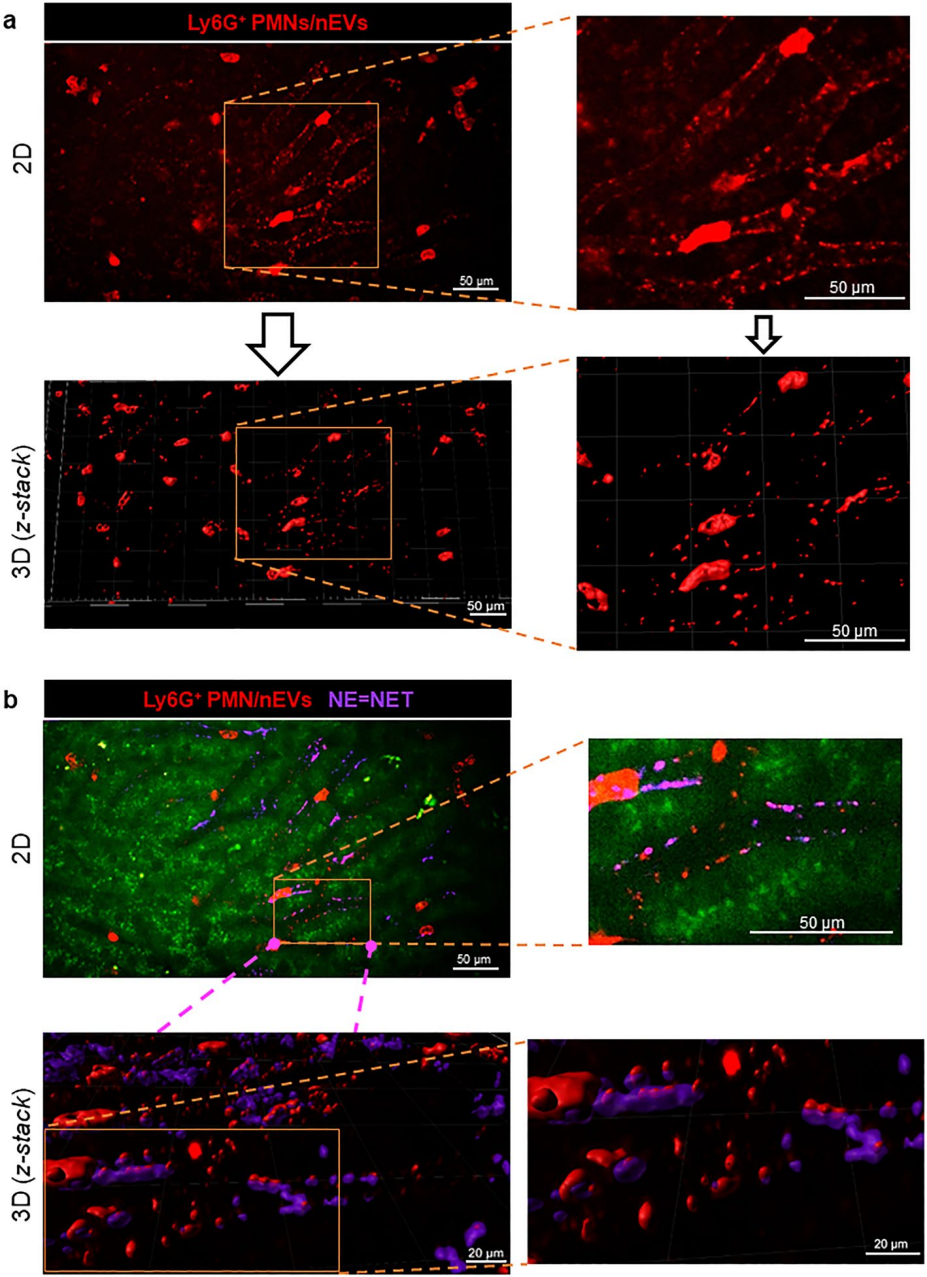
Representative images showing two-dimensional (2D) and three-dimensional (3D) images of neutrophil-derived extracellular vesicles (nEVs) and neutrophil extracellular traps (NETs) deposited in liver sinusoids. Mice with 12-h endotoxemia (LPS *i.p.* 1 mg/kg b.w.) were subjected to intravital imaging. (**a**) A single channel shows neutrophils (PMN; red cells) and nEV (red structures) obtained during 2D imaging (upper panel) and a 3D model of the same area obtained after a series of optical sections through the organ (bottom panel). (**b**) PMN, nEVs and neutrophil elastase (NE) = NET (violet) in liver sinusoids (2D, upper panel) and exemplary 3D images (bottom panel) of the same FOV visualizing co-localization of EVs with NETs (overlaying red and violet signals). Selected area of the liver was magnified to visualize microstructures in 2D ((**a**) upper panel) and 3D ((**a**) lower panel) models and co-localization of EVs and NETs in 2D ((**b**) upper panel) and 3D ((**b**) lower panel). All the images are shown from the same liver area. The photos were taken under optical magnification of 40×. The scale bar indicates 20 and 50 μm.

Since we did not detect NETs in the vascular bed of cremaster muscle (Supplementary Fig. S5biii and Fig. 2c), we focused on liver sinusoids as NET formation was well documented there in the mouse^33^. Indeed, we observed NETs in sinusoids, and they were identified by their three main components i.e., extracellular DNA, neutrophil elastase (NE), and histones (H2A.X) (Fig. 2d). Then we determined the size of each individual EVs in liver (IVM) with ImageJ software and evaluated their numbers in 5 size intervals. The size distribution of all EVs is shown in Fig. 2e, and exemplary image revealing EVs with various sizes is shown in Fig. 2f. For the identification of EVs, we made a cut at 3 μm according to the literature^7^. Larger structures were considered as EV clusters/aggregates. The same time points were followed when studying EVs in liver sinusoids as in blood plasma and peritoneal exudate. In vivo however, we focused on particular types of EVs starting with nEVs (Fig. 3). They were detected in both healthy and inflamed animals albeit in much higher numbers in the latter case (Fig. 3a). Neutrophil EVs were at first estimated as numbers of particles but their estimation by the area covered by the EV signal showed the same pattern (Fig. 3a). For this, in subsequent studies we used the area measurement as it was performed also for the NET signal (Fig. 3b). nEVs started to increase from the 6th hour of endotoxemia, reached maximum at 12 h and then drastically declined by 24 h. Along with the progression of endotoxemia also levels of NETs increased as nEVs did (Fig. 3b,c; Supplementary Fig. S6). Importantly, released EVs were present in ejected NETs and the co-localization was also highest at 12 h of inflammation (Fig. 3b,c). Additionally, both nEV secretion and NET release correlated with neutrophils infiltrating blood vessels (Fig. 3c and Supplementary Fig. S6). Moreover, the number and area of nEV aggregates (> 3 μm) in liver sinusoids were estimated (Supplementary Fig. S7) and their amount increased during endotoxemia (peak at 12 h). To better visualize EVs and their presence on endothelium and in NETs, the three-dimensional reconstruction was performed (3D; a series of optical cross-sections (z-stack)) through the mouse liver (Fig. 4a). This confirmed the co-localization of the described structures with NETs (Fig. 4a,b; Supplementary Video S3). Moreover, we observed nEV release in real time in blood vessels (Supplementary Video S4) and real time deposition of EVs in NETs (Supplementary Video S3), and interestingly, they were discharged by both nonpolarized and polarized neutrophils thus not only by highly activated cells. The latter phenomenon was independent of the health status of mice. Most EVs were attached to the endothelium (stationary) but some of them were moving along it (Supplementary Video S5). Moreover, numerous cellular interactions between neutrophils, liver macrophages–Kupffer cells (KCs), and platelets have been observed at the same time (Supplementary Video S6—Part I). Furthermore, the Supplementary Video S6—Part II shows endogenous nEVs being engulfed by KCs.

### Liver sinusoids: monocyte/macrophage-derived extracellular vesicles (mEVs) and NETs

KCs are unique to the liver^34^ and occasionally also monocytes can be detected slowing down in liver sinusoids during systemic inflammation^35^. Whereas all KCs carry F4/80 marker, only some monocytes do^35^. mEVs were detected in liver sinusoids, however, we could only detect them under 63× magnification (Supplementary Fig. S8). This was not due to EV size but because they are secreted in a wider range of levels/planes (3D) due to KC size and positioning. Indeed, at 63× magnification, mEVs were visualized at different heights of the section along the “z” axis. Levels of mEVs significantly increased at 6 h of endotoxemia and then decreased by 24 h (Supplementary Fig. S8a). Also levels of mEVs positively correlated with NETs but less of them was present in NETs (Supplementary Fig. S8a,b). All detected mEVs adhered to the vascular endothelium and no rolling was observed.

### Liver sinusoids: platelets and platelet extracellular vesicles (pEVs)

In time 0 h, fast-flowing platelets were observed in the bloodstream, but no pEVs were observed either slowing down or adhering to endothelium. Since platelets become activated very quickly during inflammation, secretion of pEVs during its early stages was additionally studied. However, no pEVs were observed at either 1 or 2 h of endotoxemia (40× optical magnification) (Supplementary Fig. S9a,b). The same results were obtained when mice were imaged at 6 h of inflammation (Supplementary Fig. S9c). Considering the above, and the small size of platelets, their rapid flow, as well as pro-coagulant properties contributing to the formation of aggregates, we visualized them under optical magnification of 63× at the 6 h of endotoxemia. However, even at this magnification, no pEVs were observed (Supplementary Fig. S9d), while numerous platelet aggregates and/or potential aggregates containing such pEVs were detected (Supplementary Fig. S9e).

### Effect of PAD4 inhibitor on EV secretion and NET formation

**Figure 5:**
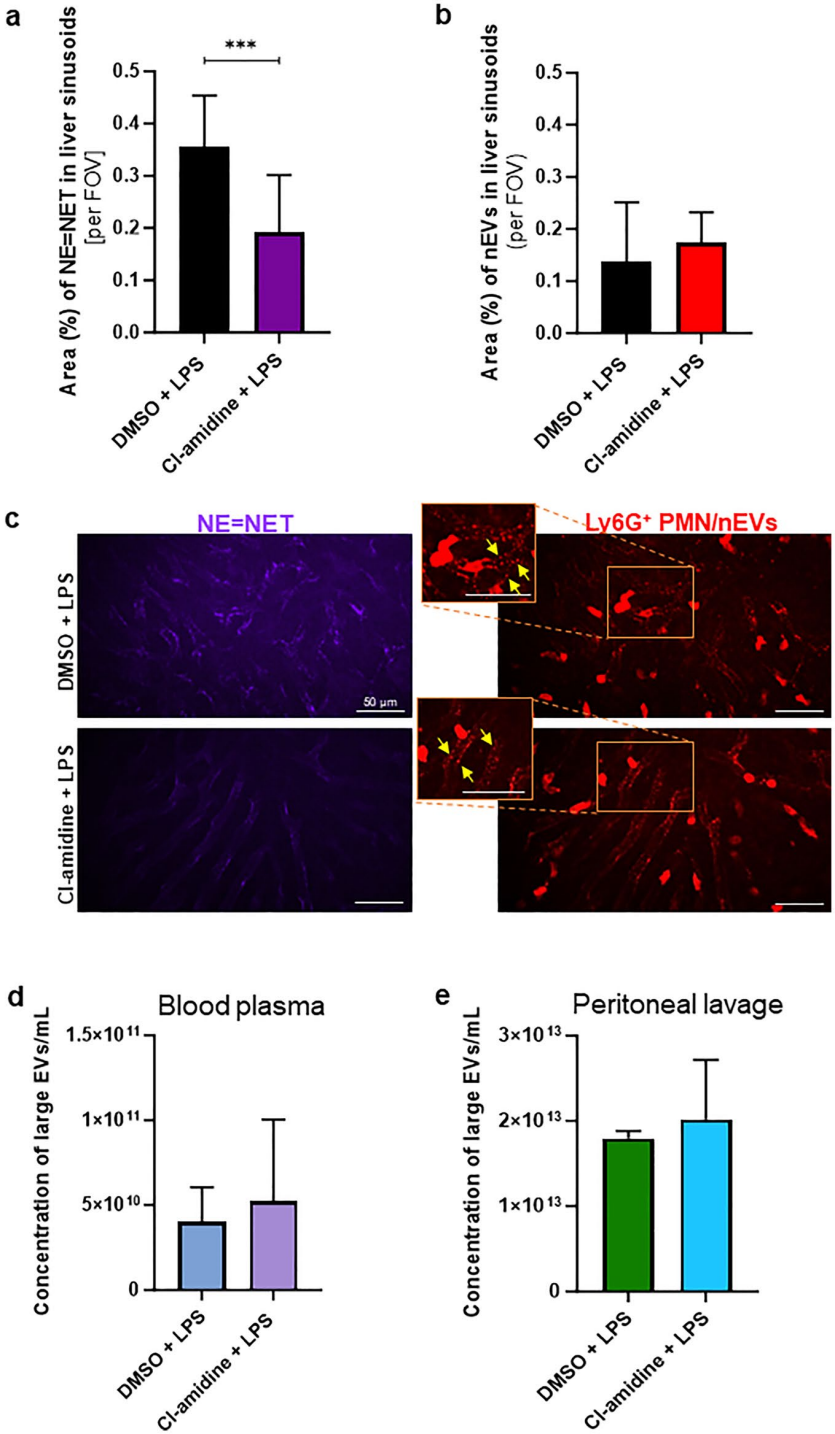
Impact of NET inhibition on extracellular vesicles (EVs) and neutrophil extracellular trap (NET) formation/release in endotoxemic mice**.** C57BL/6 J mice with lipopolysaccharide (LPS)-induced endotoxemia (*i.p.* 1 mg/kg b.w.; 8 h post LPS inoculation) were used in the study. Some mice were *i.p.* injected with 5% DMSO or Cl-amidine (40 mg/kg b.w.) 1 h prior to endotoxemia induction. Animals were imaged or samples were collected 8 h later. (**a**–**c**) The liver sinusoids were imaged in vivo by Spinning Disk Confocal Intravital Microscopy (SD-IVM), and (**d**,**e**) EVs were analyzed with the nanoparticle tracking analysis (NTA) system. Area of (**a**) NE = NET and (**b**) EVs in liver sinusoids. (**c**) Representative images of NETs (NE) and neutrophil-derived EVs (nEVs) formed in response to LPS with DMSO (upper panel) and Cl-amidine (bottom panel). The images were taken under optical magnification of 40×. The scale bar indicates 50 μm. Concentration of EVs in (**d**) blood plasma and (**e**) peritoneal lavage. The results are expressed as the mean values ± SD. Data were analyzed with Mann–Whitney test (panels (**a**,**b**,**d**)) or unpaired two-tailed Student’s *t*‐test (panel (**e**)). Asterisks indicate significant differences using Mann–Whitney test ***p ≤ 0.001; n = 3. FOV—field of view.

Knowing that EVs co-localize with ejected NETs, we checked whether one of the studied structures (NET) affects the other (EV). The use of PAD4 inhibitor (Cl-amidine) which prevents histone citrullination preceding NET formation, indeed inhibited NETs but did not affect EV secretion (Fig. 5a–e). In particular, no changes in EVs were observable on liver endothelium (Fig. 5b,c) and this was confirmed by NTA for EVs isolated from blood plasma (Fig. 5d) and peritoneal lavage (Fig. 5e).

### Exogenously labeled EVs are taken up by Kupffer cells but some are captured by NETs

**Figure 6:**
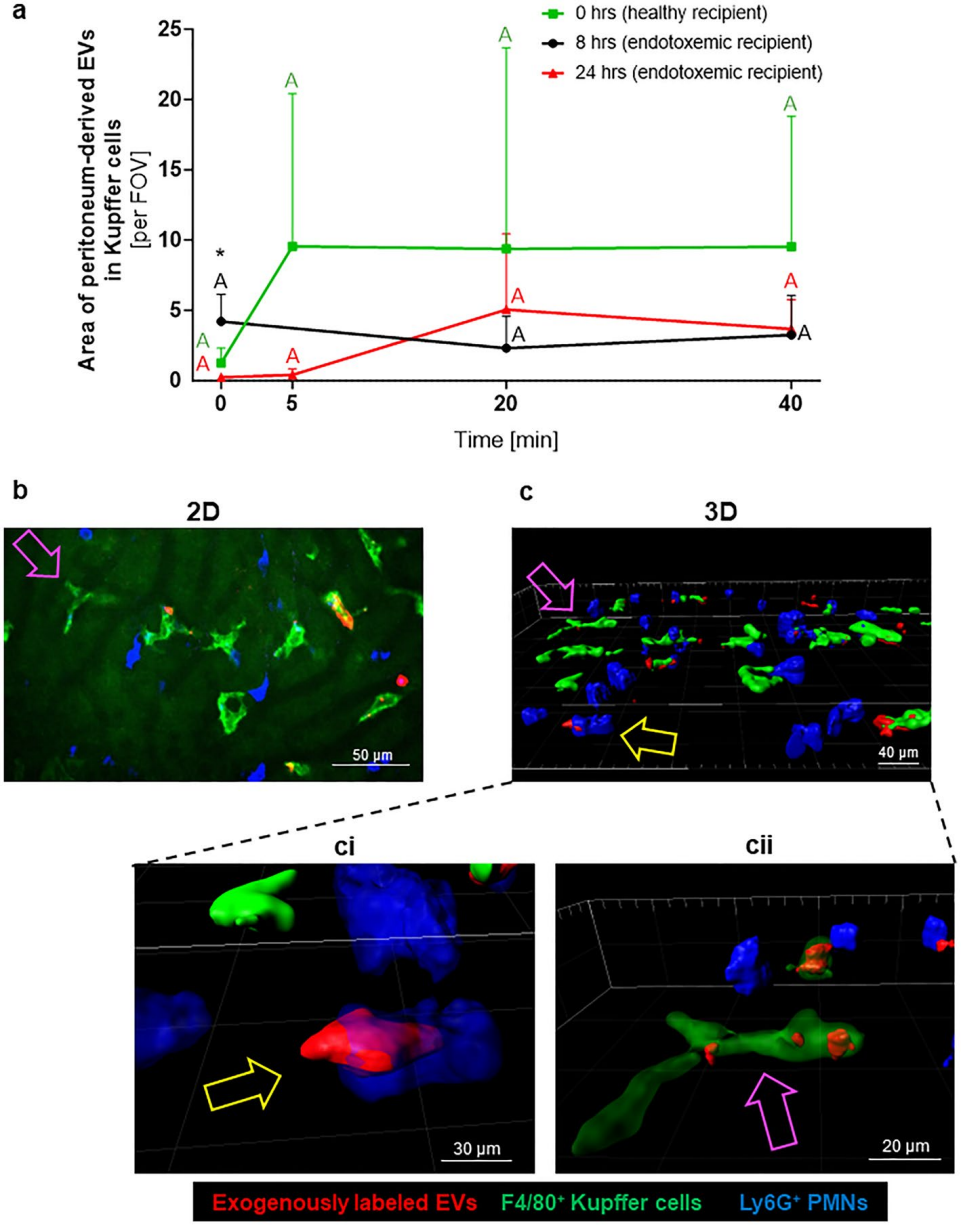
Exogenously labeled extracellular vesicles (EVs) in liver sinusoids. Exogenously labeled EVs obtained from peritoneal lavage of C57BL/6 J mice with lipopolysaccharide (LPS)-induced endotoxemia (*i.p.* 1 mg/kg b.w.; 8 h post LPS inoculation) were used. They were *i.v.* injected into healthy mice (0 h) and mice with 8- and 24-h endotoxemia which were subsequently subjected to in vivo imaging with Spinning Disk Confocal Intravital Microscopy (SD-IVM). (**a**) Exogenous EVs derived from peritoneal lavage were detected inside of hepatic macrophages (Kupffer cells) independently of the recipient mouse (healthy—0 h or 8/24-h endotoxemia). The EV area was estimated at selected time points (0, 5, 20 and 40 min) after injection of exogenous EVs. Values significantly different (*p˂0.05*) according to one-way ANOVA with Bonferroni multiple comparisons post hoc test are designated by letters (different letters indicate statistical differences). Asterisks indicate statistically significant differences (between the study groups at particular time points) according to unpaired two-tailed Student's t-test (*p ≤ *0.05*). (**b**,**c**) Representative images showing two- (2D) and three-dimensional (3D) images of exogenous EVs (red) in liver sinusoids. Additionally, neutrophils (blue cells) and Kupffer cells (bright green cells against a background of dim green autofluorescent hepatocytes) were stained (**b**) 2D image and (**c**) 3D image (a series of optical sections through the liver; z-stack). EVs present inside of leukocytes are indicated by arrows, e.g., the pink arrow points to the same macrophages whose colors in 2D correspond to specific cells in 3D. The enlarged parts of the 3D model show semi-transparent (**ci**) neutrophil and (**cii**) macrophage to visualize the EVs inside the cells. All images shown were from the same imaged liver site. The photos were taken under optical magnification of 40x. The scale bar indicates 20, 30, 40 or 50 μm; n = 3. FOV—field of view.

Subsequently we attempted to verify if EVs impact NET formation. EVs were isolated from blood plasma and peritoneal lavage of mice with endotoxemia (at 8 h), and then they were exogenously stained with a dye staining the cytoplasmic component of EVs. Stained EVs were then intravenously injected into healthy mice (0 h) and animals with 8- and 24-h ongoing endotoxemia during continuous intravital imaging. IVM detected the injected EVs (Fig. 6, Supplementary Figs. S10 and S11), however, within the first 5 min the majority of them were phagocytized by either KCs (bulk) or neutrophils (some) (Fig. 6a–c and Supplementary Fig. S10a). EVs could also be detected attached to endothelium, some were present inside hepatocytes or in NETs. Because of the fast engulfment of exogenous EVs, we were unable to beyond doubt assess their impact on NET formation. We did see much less NETs (Supplementary Fig. S12) but we were unable to estimate the cause of it. It could have been triggered by EVs, KCs phagocytosis, injection itself thus we switched to the ex vivo system to clarify it (next paragraph). In regard to the remaining in vivo observations, levels of non-phagocytosed EVs did not change during the 40-min observation (Supplementary Figs. S10b,c and S11a,b). A sequence of optical cross-sections aimed to reconstruct 2D images to the 3D version confirmed the presence of exogenously labeled EVs inside leukocytes (Fig. 6b,c). The semi-transparent mode revealed EVs inside neutrophils and macrophages (Fig. 6ci,cii).

### EVs affect NETs ex vivo

**Figure 7:**
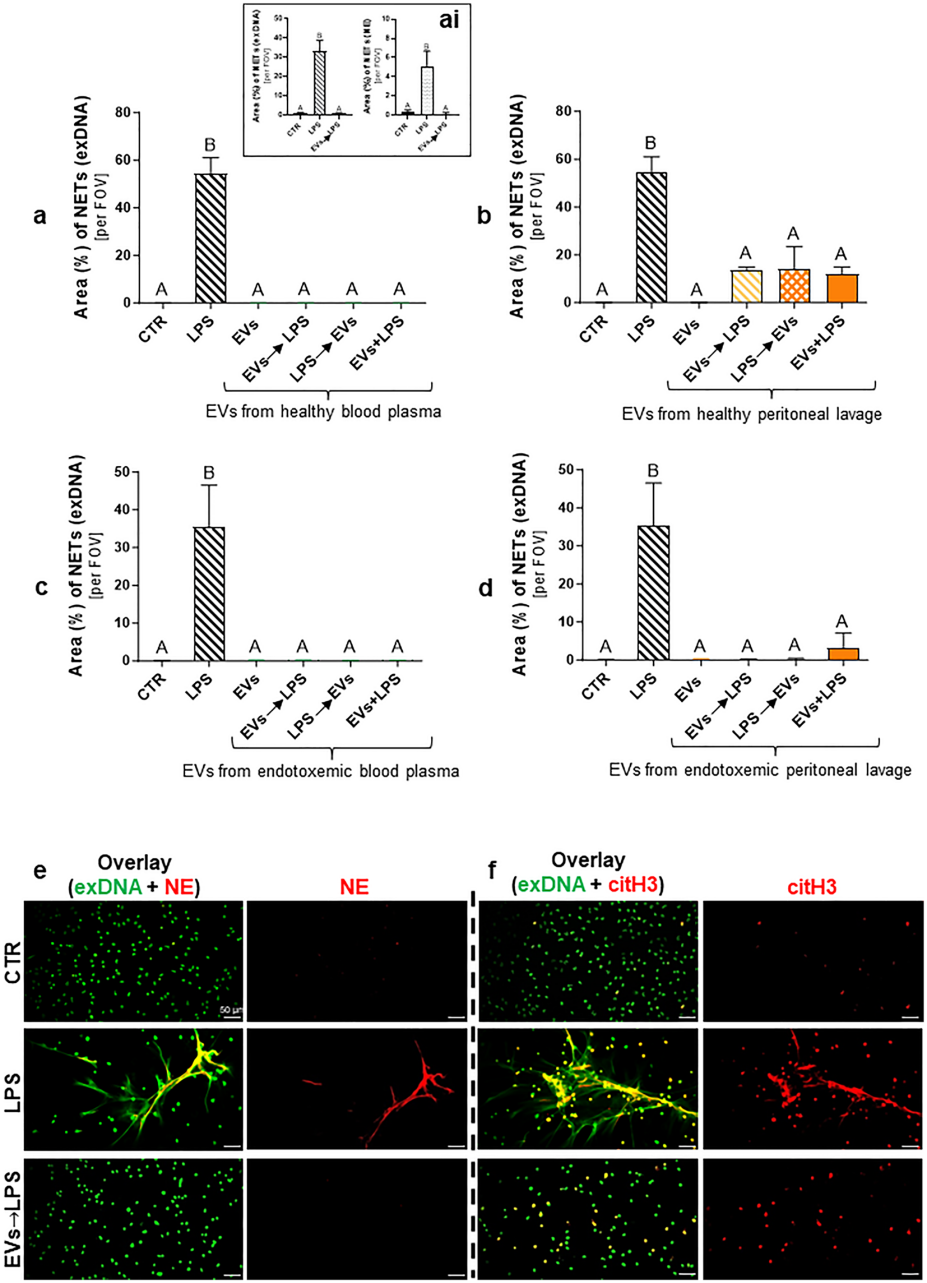
Impact of extracellular vesicles (EVs) isolated from blood (plasma) and peritoneal lavage on the neutrophil extracellular traps (NETs) release. Extracellular vesicles were isolated from blood plasma and peritoneal lavage of healthy (0 h) C57BL/6 J mice and mice with lipopolysaccharide (LPS)-induced endotoxemia (*i.p.* 1 mg/kg b.w.; 8 h post LPS inoculation). The obtained EVs were added to the neutrophils isolated from the bone marrow for a 6-h incubation. The following groups were tested: unstimulated cells (CTR); LPS-stimulated cells (75 μg/mL); EVs at a density of 50 × 10^6^ EV/10^5^ cells; EVs at a density of 50 × 10^6^ EVs/10^5^ cells (30 min) before adding LPS (EVs → LPS); cells stimulated with LPS (30 min) before adding EVs (LPS → EVs); simultaneous administration of EVs with LPS (EVs + LPS). Area (%) occupied by NETs (exDNA) obtained from (**a**,**ai**) blood plasma and (**b**) peritoneal lavage of healthy mice and (**c**) blood plasma and (**d**) peritoneal lavage of mice with endotoxemia. (**ai**) Comparison of data quantification for exDNA with neutrophil elastase (NE) signal. The results are expressed as the mean values ± SD. Values significantly different (*˂0.05*) according to Kruskal–Wallis's test followed by Dunn’s test (panels c,d) or one-way ANOVA with Bonferroni multiple comparisons post hoc test (panels a,b) are designated by letters (different letters indicate statistical differences); n = 3 (3 repetitions). FOV—field of view. Representative images of NETs formed in response to EVs (EV → LPS) obtained from blood plasma of healthy mice visualized by co-staining of (**e**) extracellular DNA (green) with NE (red), and (**f**) extracellular DNA (green) with citrullinated histone (citH3; red). To visualize co-localization of NET components, the images from each channel were overlaid. The photos were taken under optical magnification of 20×. The scale bar indicates 50 μm.

We continued studies on the impact of EVs on NET formation on isolated cells and EVs obtained from blood plasma and peritoneal lavage and they significantly inhibited NET formation by neutrophils isolated from bone marrow (Fig. 7). The inhibition was most dramatic in the case of blood plasma EVs and was independent of the timing of LPS addition (before, after or together with EVs) or their origin from healthy donors or endotoxemic mice (Fig. 7a,c,e,f). Although inhibition of NET formation was also detected in the presence of peritoneal EVs, this effect was less profound and much stronger if EVs were isolated from endotoxemic mice (Fig. 7b vs. d).

### Identification of the origin of EVs affecting NET release

**Figure 8:**
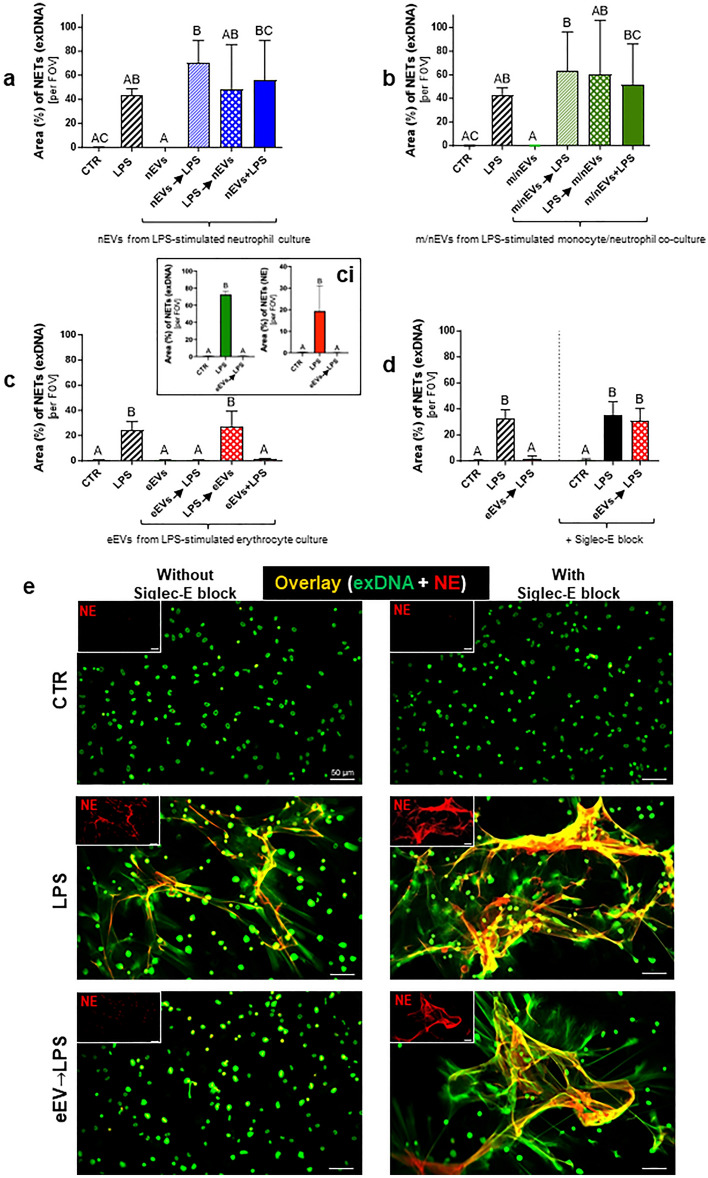
Impact of extracellular vesicles (EVs) isolated ex vivo from neutrophil and erythrocyte cultures and neutrophil/monocyte co-culture on the neutrophil extracellular traps (NETs) release. Extracellular vesicles were collected from previously isolated neutrophils, monocytes from the bone marrow, while erythrocytes were obtained from blood of healthy (0 h) C57BL/6 J mice. The isolated cells were stimulated ex vivo with lipopolysaccharide (LPS; 75 μg/mL; 4-h incubation) to induce the secretion of EVs. The obtained EVs (cell supernatant) were added to the neutrophils isolated from the bone marrow (6-h incubation). The following groups were tested: unstimulated cells (CTR); LPS-stimulated cells (75 μg/mL); EVs at a density of 50 × 10^6^ EVs/10^5^ cells; EVs at a density of 50 × 10^6^ EVs/10^5^ cells (30 min) before adding LPS (EVs → LPS); cells stimulated with LPS (30 min) before adding EVs (LPS → EVs); simultaneous administration of EVs with LPS (EVs + LPS). In some treatments prior (30 min) to EV and LPS stimulation (EVs → LPS), neutrophils were incubated with the Siglec-E blocker (diluted 1:200). Area (%) occupied by NETs (exDNA) released from neutrophils under the influence of EVs stimulation obtained from (**a**) cultures of neutrophils, (**b**) co-cultures of neutrophils with monocytes, and (**c**,**ci**) cultures of erythrocytes, and (**d**) in response to eEVs (eEVs → LPS) obtained from erythrocytes after previous incubation of cells with the Siglec-E blocker. (**ci**) Comparison of data quantification for exDNA with neutrophil elastase (NE) signal. The results are expressed as the mean values ± SD. Values significantly different (*p˂0.05*) according to Kruskal–Wallis's test followed by Dunn's test (panels a,b,d) or one-way ANOVA with Bonferroni multiple comparisons post hoc test (panel c,ci) are designated by letters (different letters indicate statistical differences); n = 3 (3 repetitions). FOV—field of view. (**e**) Representative images of NETs formed in response to eEVs without (left panel) or with (right panel) the Siglec-E blocker, visualized by co-staining of extracellular DNA (green) with NE (red). To visualize co-localization of NET components, the images from each channel were overlaid. The photos were taken under optical magnification of 20×. The scale bar indicates 50 μm.

Isolated neutrophils, monocytes (80% purity) or erythrocytes were stimulated ex vivo with LPS, then the supernatant was collected and nEVs, m/nEVs and eEV were isolated. They were subsequently used to stimulate neutrophils alone or in combination with LPS (Fig. 8). Neither neutrophil (Fig. 8a) nor monocyte/neutrophil (Fig. 8b) EVs significantly affected NET formation in any configuration. In contrast, eEVs when added prior to LPS or together with it completely blocked NET release (Fig. 8c,ci). This was not the case only when neutrophils were at first exposed to LPS and eEVs added 30 min later (Fig. 8c).

### Inhibition of NET release by eEVs depends on Siglec-E

Pretreatment of neutrophils with the Siglec-E blocker successfully inhibited Siglec-E (Supplementary Fig. S13). When the blocking antibodies were applied prior to EVs and LPS stimulation they completely prevented the inhibition of NET formation i.e., NET formation was restored (Fig. 8d,e).

## Discussion

EV secretion and NET casting are commonly observed in pathological conditions, including systemic inflammation^17,36^. They are seemingly well-understood structures in terms of formation and basic functions yet their mutual interactions/dependency, and thus overall impact on pathological conditions, remain mostly unknown. Herein we report four major findings—(i) time-dependent release of EVs in real time as it occurs in the vasculature of mice, (ii) interplay between EVs and NETs in which the former impact the traps but not vice versa*,* and we reveal (iii) composition of EVs retrieved from blood differs from those collected from the peritoneal lavage and is not representative of EVs interacting with leukocytes and NETs on endothelium in vivo*.* And last but not least (iv) we show that EVs released by erythrocytes inhibit NET formation in a Siglec-E-dependent manner.

During endotoxemia we detected that large EV secretion into blood and peritoneal lavage increased as reported previously e.g. in trauma patients or those with severe systemic inflammatory response syndrome (SIRS)^37,38^. In contrast, kinetics of small EVs did not strictly correlate with the course of endotoxemia, however, their involvement in numerous other conditions was clearly demonstrated^39^. For the above reasons we focused on large EVs, especially that we could detect them under the confocal microscope unlike small ones.

Whereas pEVs dominated in blood, in the peritoneal lavage these were mEVs. These data imply that the dominating source of EVs relates to quantity of populations in a given body compartment. In terms of numbers, erythrocytes are most numerous in blood but platelets are the second most frequent elements, and so are resident macrophages in the peritoneal cavity of mice^40,41^. Similar results were obtained when analyzing blood of patients with septic shock^42^.

Subsequently we focused on the visualization of EVs in vivo. At first, we established their detection by identifying the source cell (surface markers) and size. Not only did we image such structures adhering to endothelium or rolling along it but we also captured the release of vesicles with such characteristics from leukocytes confirming our criteria. They do resemble a subtype of EVs described previously as migrasomes^43,44^. Migrasomes are hypothesized to enhance the immune surveillance system by neutrophils. Moreover, in studies in which we at first isolated EVs from plasma or peritoneal cavity, pre-stained them and injected back into mice, these structures appeared (shape- and size-wise) as endogenous EVs once in the vasculature. Those that were not engulfed by phagocytes, also behaved in the same manner.

EVs described above were detected in two distinct blood vessels of cremaster muscle and liver sinusoids. The former represents a classical vascular bed whereas sinusoids have unique characteristics and structure, with highly specialized liver sinusoidal endothelial cells (LSEC)^45,46^. It should be stressed that thus far migrasomes were described only in liver sinusoids^43,44^. During homeostasis we did not capture any nEVs in the vessels of cremaster muscle whereas during endotoxemia, numerous neutrophils infiltrated blood vessels, rolling or adhering to endothelium, and their numbers positively correlated with nEVs adhering to the vascular lining. These findings concur, and further extend, previous studies on neutrophil trails leaving bioactive EVs^9–11^, ENDS released from neutrophil microvilli^8^ or uropods formed by extravasating leukocytes^9^. Clearly, neutrophils generate different types of EVs in various immunologic milieu. In the liver we systematically imaged nEV release in real time by both non-polarized (hemostasis) and polarized (homeostasis/inflammation) neutrophils. Polarized neutrophils secreted EVs during the rolling and crawling along the endothelium. EVs released from non-polarized neutrophils in healthy mice rolled over endothelium whereas during endotoxemia EVs immediately adhered to endothelium. This indicates activation of the latter, characteristic for systemic inflammation^47^. Importantly, nEVs interacted with neutrophils, KCs and platelets. Some nEVs were visible inside liver macrophages indicating their uptake. Thus far the intake of EVs adhering to liver sinusoids was only shown by other neutrophils^44^.

Because NETs were not detectable in the cremaster, we continued imaging EVs in the liver where trap formation was shown previously^21,33,48^. NET detection (IVM) in the liver sinusoids but not in other organs (such as lungs^49^) results probably from slower blood flow therein and a delicate nature of NET structure^50^. Moreover, liver populated by KCs is a site of pathogen entrapping^48^.

When studying concomitantly EVs and NETs we detected that about half of visible particles was trapped in NETs, and their release peaked at the same time. Moreover, we captured the moment when a released EV bound directly to NETs. Interactions between EVs and NETs visualized using IVM are consistent with previous ex vivo reports^26^. In particular, isolated stimulated neutrophils were shown to eject NETs and generate EVs, some of which were immobilized in the traps^26^.

Neutrophils are dominating leukocytes engaged in systemic inflammation whereas blood monocytes synthesize cytokines during early stages of sepsis and later down-regulate inflammatory genes^51^. In contrast, resident macrophages such as KCs are engaged at all stages of inflammation^52,53^. In this study, the presence of mEVs in plasma, and peritoneal lavage of healthy and endotoxemic animals was demonstrated by flow cytometry and IVM. Also, mEVs co-localized with NETs which might indicate a lack of preference for the binding of EVs of specific cellular origin. mEVs were attached to endothelium, and we did not capture their movement nor release in real time. This might indicate that they are less reactive with endothelium and less frequently released hence lower chance of capturing the process, respectively. The most surprising was absence of single pEVs on endothelium. They were clearly released by platelets as detected by flow cytometry and also others showed that they constitute the largest population of vesicles^54^. Moreover, we observed multiple CD49^+^ aggregates, however, we cannot exclude these are platelet/pEV aggregates. These clusters of platelet-related structures were mostly detected on either KCs or neutrophils, a phenomenon described in detail previously^55,56^, but not on endothelium.

Knowing that EVs co-localize with NETs, we checked whether NETs affect their secretion in vivo. We inhibited NET formation but this did not change EV numbers present in liver sinusoids. Neither NTA registered any difference in EVs in blood or peritoneal lavage. These results are consistent with the studies of Wang et al.^26^, who showed that the co-incubation of isolated neutrophils with DNase and NET inhibition did not affect EVs secretion^26^. To verify whether the EV-NET interaction is bi-directional, fluorescently-labelled EVs were administered into recipient mice. Their vast majority was phagocytized within first 5 min by KCs, and to a lesser extent by neutrophils. KCs are indeed efficient in capturing foreign bodies^21^ and EV phagocytosis was observed before^57,58^. The few unphagocytized EVs co-localized with NETs demonstrating that they bind to or are caught up by NETs. But to further study if EVs affect NET formation we had to switch to isolated neutrophils. We also tested if the timing of vesicle addition is of importance i.e., if the inflammatory insult (LPS) has to occur first or not. EVs originating from plasma almost completely inhibited NETs even those isolated form healthy mice. In the case of peritoneal EVs, such a strong effect was observed only when they originated from endotoxemic mice. This effect could be just cargo-dependent^59^, however, could also relay on surface molecules. We subsequently identified RBCs as the source of EVs that inhibit NET formation. Involvement of erythrocytes in immune responses was identified only recently^60^, and the cells were shown to inhibit NET formation via interaction between their surface glycophorin A and its ligand (Siglec-9) on neutrophils^61^. Herein we show for the first time that eEVs themselves have the same capacity. Considering that only few erythrocytes are present in the peritoneal cavity, especially in homeostasis, this might explain weaker impact of peritoneal vesicles on NETs. Interestingly, eEVs did not exert their inhibitory effect when applied after LPS whereas the whole population of EVs did. It might be that once neutrophils are already primed to release NETs it is too late to stop the process and/or there might be another population involved in the process that was not identified yet. Thus far only stimulation of NET formation was shown by EVs^24,28,62^.

The presented data show that the use of intravital microscopy allows for visualization of leukocyte EVs in real time in their natural environment. The study reveals that EVs are bioactive structures that affect/modulate NET release which role in systemic inflammation is largely detrimental. In particular EVs released from erythrocytes inhibit NETs confirming their recently recognized role in immunomodulation. EV interactions with NETs are not bidirectional, indicating no effect of NETs on EV secretion. In addition, our study demonstrated that the results obtained by IVM and flow cytometry are complementary and should be conducted in parallel to form the whole picture of the complex nature of EVs, a significant example of which is the low detection of circulating nEVs in the blood and their increased number captured on liver and cremaster muscle vascular endothelium.

## Materials and methods

### Mice

C57BL/6 J male mice (3–6 weeks old) were purchased from the Charles River Laboratories (Sulzfeld, Germany; via AnimaLab). Mice were used in experiments at the age of 8–12 weeks old. Mice were housed in standard environmental conditions and had access to commercial food and tap water (both ad libitum*).* All animal procedures and relevant experiments/methods were performed in accordance with Guide for the Care and Use of Laboratory Animals (Directive 2010/63/EU of European Parliament), Polish legislation (Act 266/2015; updated in 2021) and institutional animal care policies. The animal experimental protocols for the study were approved by the Local Ethical Committee No. II in Krakow (294/2017 and 244/2019). Animal use followed the recommendations of the ARRIVE guidelines.

### Induction of endotoxemia/systemic inflammation

Mice were intraperitoneally (*i.p*.) injected with 1 mg/kg per body weight (b.w.) lipopolysaccharide (LPS, *Escherichia coli,* serotype 0111:B4; Sigma‐Aldrich, Saint Louis, MO, USA) in 0.9% saline (NaCl) to induce endotoxemia^48^. Mice were subsequently subjected to sample collection or intravital imaging as detailed below. Some of their counterparts were untreated (healthy controls, 0 h).

### Cl-amidine: PAD4 inhibitor

To inhibit NET formation, mice were injected intraperitoneally with the PAD4 inhibitor Cl-amidine (40 mg/kg b.w. in 5% DMSO; Sigma-Aldrich, Saint Louis, MO, USA) 1 h before induction of endotoxemia (8 h). The control consisted of mice injected only with the solvent—5% DMSO. Blood and peritoneal lavage were collected for further determinations or mice were subjected to in vivo imaging.

### Preparation of liver for intravital microscopy (IVM)

Mice were anesthetized with a mixture of ketamine hydrochloride (200 mg/kg b.w.; Biowet Pulawy, Pulawy, Poland) and xylazine hydrochloride (10 mg/kg b.w.; aniMedica, Südfeld, Germany). Subsequently cannulation of the right jugular vein was performed to administrate anesthetics/antibodies or other dyes. Preparation of the liver for IVM was performed as previously described^63^.

### Preparation of cremaster muscle for IVM

Mice were anesthetized with a mixture of ketamine and xylazine hydrochloride as described above. Subsequently cannulation of the right jugular vein was performed to administrate anesthetics/antibodies or other dyes. Preparation of the cremaster muscle for IVM was performed as previously described^56,63^. Briefly, mice were placed on the Plexiglas board dedicated for cremaster muscle. A small piece of the scrotum was cut to expose the left testis from which cremaster muscle was carefully extracted. Then, the muscle was cleaned of connective tissue, and separated from the testis by thermal cutting (with a cautery) of the thin ligaments connecting the muscle with the testis. Visualized cremaster muscle was placed on a previously prepared optically clear board. The testis and epididymis were backed inside the abdominal cavity.

### Spinning disk confocal intravital microscopy (SD-IVM)

Both vascular beds–sinusoids of the liver and cremaster muscle were imaged with a ZEISS Axio Examiner.Z1 upright microscope equipped with a metal halide light source (AMH-200-F6S; Andor, Oxford Instruments, Abingdon, UK) with motorized 6 position excitation filter wheel and laser-free confocal spinning disk device (DSD2; Andor, Oxford Instruments, Abingdon, UK) with ZEISS EC Plan-NEOFLUAR 10 × /0.3, ZEISS EC Plan-NEOFLUAR 20 × /0.5, ZEISS LD Plan-NEOFLUAR 40 × /0.6 and 63 × /0.75 air objective. The following filters were used, four excitation filters (DAPI: 390/40 nm; GFP: 482/18 nm; RFP: 561/14 nm; Cy5: 640/14 nm) and appropriate emission filters (DAPI: 452/45 nm; GFP: 525/45 nm; RFP: 609/54 nm; Cy5: 676/29 nm). For fluorescence detection, the 5.5-megapixel sCMOS camera (Zyla 5.5; Andor, Oxford Instruments, Abingdon, UK) was used and the iQ 3.6.1 acquisition software (Andor, Oxford Instruments, Abingdon, UK) to drive the microscope.

### 3D reconstruction of liver cross-sections

A series of optical cross-sections (z-stacks) through the mouse liver was used to obtain 3D reconstruction (IMARIS v8.4.2 software, Bitplane, Oxford Instruments, Abingdon, UK). It took a series of 50–70 images in the overall range of up to 100 μm in the “z” plane. A series of images were taken separately for each fluorescence channel (depending on the experimental setup: GFP, RFP, DAPI, and/or Cy5), and then all channels were merged into one single image. In addition, in some experiments, to confirm the co-localization of EVs with NETs and intracellular presence of EVs, semi-transparency (translucency) of cells was selected in the IMARIS software.

### Visualization of leukocyte/platelet-derived extracellular vesicles (EVs) and leukocytes with IVM

Neutrophils and neutrophil-derived EVs (nEVs) were stained with anti-mouse Ly6G antibodies (1.6 ug/mouse; PE, Brilliant Violet 421 or AlexaFluor 488 anti-Ly6G, clone 1A8, BioLegend, San Diego, CA, USA) and Kupffer cells (KCs)/Kupffer cells-derived EVs or monocytes/macrophages-derived EVs (mEVs) were visualized with PE, AlexaFluor 488 or eFluor 660 conjugated anti-F4/80 monoclonal antibodies (1.2 μg/mouse, clone BM8; eBioscience, San Diego, CA, USA). Platelets were visualized with anti-CD49b antibodies (1.2 μg/mouse; PE anti-CD49b, clone HMα2, BioLegend, San Diego, CA, USA). Endothelial cells were stained with anti-CD31 (PECAM) antibodies (5 μg/mouse; Alexa Fluor 488 anti-CD31, clone 390, BioLegend, San Diego, CA, USA). Cells and EVs were visualized at magnification of 20, 40 or 63 ×. Numbers of EVs were counted under 40× magnification, minimum 3 fields of view (FOVs) from each mouse.

### Visualization and analysis of NETs: in vivo

NET imaging was performed by intravital immunofluorescence analysis. NET components were visualized by co-staining of histones H2A.X (0.5 µg/mouse; Alexa Fluor 555 anti-H2A.X antibody, clone 938CT5.1.1, Santa Cruz Biotechnology, Dallas, TX, USA), neutrophil elastase (1.6 μg/mouse; Alexa Fluor 647 anti-neutrophil elastase antibody, clone G-2, Santa Cruz Biotechnology, Dallas, TX, USA), and extracellular DNA (0.1 mM in 0.9% saline; Sytox green, Invitrogen, Carlsbad, CA, USA). The antibodies were administered intravenously (via the jugular vein) approx. 20 min prior to intravital imaging of the liver. Sytox green was administrated during liver imaging. The estimation of the NE area was performed using the ImageJ v 1.53 s software (National Institutes of Health, Bethesda, MD, USA). The obtained images from in vivo imaging were converted to a binary image—16-bit type, and the positive fluorescence signal (black on white) from neutrophil elastase was then estimated. Quantitative data were expressed as a percentage (%) of the area in each FOV covered by positive fluorescence staining.

### Estimation of the number/area of neutrophil-derived extracellular vesicles (nEVs) and their aggregates and EVs of monocyte/macrophage (mEVs) origin: in vivo

The number of EVs and area covered by EVs as well as area of EV aggregates were estimated from the obtained images which were converted to a 16-bit type image using ImageJ software. The positive signal (black on white) from EVs presents in the blood vessels of liver and/or vasculature of cremaster muscle was estimated. Numbers of EVs were calculated according to von Aspern et al.^64^. Briefly, the following formula was used to estimate EV numbers: ø = 2 × √(A/π), where ø is a diameter and A an area of each EV. Only structures ≤ 3 µm in size where counted. In majority of studies, EVs were estimated by area covered by them. This was due to the aggregation properties of EVs, and to confine to the same parameter (“% of area”) as in the case of NETs. The number and area of EVs were counted/estimated under magnification of 40 and/or 63 × in minimum 3 FOVs from each mouse.

### Estimation of co-localization EVs with NETs

The overlapping positive signals (black on white) from EVs and neutrophil elastase (NETs) were used to estimate their co-localization. Results are expressed as percentage of co-occupied area per FOV. The co-localization area was estimated from minimum 3 FOVs from each mouse.

### Sample collection for extracellular vesicles (EVs) retrieval

Mice were anesthetized with a ketamine/xylazine mixture and subjected to blood and peritoneal lavage collection. Blood (approx. 1 mL) was collected from each mouse by cardiac puncture in a heparinized syringe, and peritoneal lavage/exudate was collected by lavage of peritoneal cavity with 1.5 mL of saline, and after 30 s gentle manual massage peritoneal lavage/exudate was retrieved. Samples were centrifuged at 400 *g* and 1200 *g* consecutively for 10 min at room temperature (RT), and then at 1500 *g* for 15 min at RT for the retrieval of debris-free plasma and peritoneal lavage. Both blood plasma and peritoneal lavage were collected at the selected time-points of endotoxemia as indicated on the graphs, and were immediately subjected to the EV isolation procedure. Mice were euthanized by overdosing of ketamine/xylazine mixture.

### Extracellular vesicles isolation and ex vivo analysis

EVs were isolated as described previously^65^. The collected supernatant from cell/debris-free plasma and peritoneal lavage was pelleted in a centrifuge twice at 14,000 *g* for 35 min at 4 °C to obtain large EVs. Supernatant from large EVs pellet was used for smaller EVs isolation—it was centrifuged at 100,000 *g* for 90 min at 4 °C (Sorvall WX + Ultracentrifuge Series). Before centrifugation, large EVs and small EVs pellets were washed with sterile 1 × PBS and were analyzed with Nanoparticle Tracking Analysis (NTA) using NanoSight system—LM10HS microscope equipped with the LM14 488 nm laser module (Malvern Instruments Ltd., Malvern UK). Filtered PBS was used for setting the threshold to eliminate instrument noise (Supplementary Figs. S2e and S3a). The size, distribution and movement of blood plasma- and peritoneal lavage-derived EVs were determined by NTA which tracks in real time each particle individually in liquid suspension, using both Brownian motion and light scattering^66^. For this purpose, samples with EVs were diluted 500 times in prefiltered (0.2 μm), sterile 1 × PBS to the total volume of 1 mL. Subsequently, solution was placed into an insulin syringe and loaded into the sample chamber. The EVs concentration measurements were taken from 60 s video recordings captured by the sCMOS camera and calculated using the NanoSight NTA 2.3 analytical software (Malvern Instruments). Representative examples of NTA histograms are presented in Supplementary Fig. S2.

### Imaging of extracellular vesicles with transmission electron microscope (TEM)

Visualization of EVs was performed with a negative-stain transmission electron microscope (TEM) coated with formvar, with 300 mesh copper grids prepared for each EV sample using 2% uranyl acetate (Chemapol, Prague, Czech Republic). For observation, the JEOL JEM 2100HT transmission electron microscope (Jeol Ltd, Tokyo, Japan) was used at accelerating voltage 80 kV. Images were taken by using 4k × 4k camera (TVIPS) equipped with EMMENU software ver. 4.0.9.87.

### Exogenous (ex vivo) labeling of EVs

After anesthesia, blood plasma and peritoneal lavage were collected from 5 endotoxemic mice (8 h) for EVs isolation (as described above). Then EVs were stained with CellTracker™ Red CMTPX (Invitrogen, ThermoFisher Scientific, Waltham, MA, USA) according to the manufacturer's guidelines. Briefly, collected EVs were incubated at 37 °C for 40 min with 1 × PBS containing 5 μM cell tracker dye. After this time the unbound dye was removed (14,000 *g*, 20 min at RT) and EVs were suspended with 200 μL NaCl. After the labeling procedure, EVs (at a concentration of 6.5 × 10^11^ per mouse) were administered intravenously to healthy (0 h) and endotoxemic (with 8- or 24-h ongoing inflammation) recipient mice during ongoing IVM imaging. The fate/biodistribution of EVs in the blood vessels of mice was followed for 40 min after their administration.

### Flow cytometric analyses of EVs of various origin

Employing flow cytometry (FACSCanto, BD Biosciences Immunocytometry Systems, San Jose, CA, USA), EVs isolated from blood plasma and peritoneal fluid were gated as neutrophil-derived Ly6G^+^ EVs (PE anti-mouse Ly6G, 1A8, BioLegend, San Diego, CA, USA), monocyte/macrophage-derived F4/80^+^ EVs (PE anti-mouse F4/80, BM8; eBioscience, San Diego, CA, USA), platelet-derived CD41^+^ EVs and CD62P^+^ EVs (PE anti-mouse CD41, MWReg30 and PE anti-mouse/rat CD62P, RMP-1, both BioLegend, San Diego, CA, USA) as well as erythrocyte-derived TER-119^+^ EVs (PE anti-mouse TER-119, TER-119, BioLegend, San Diego, CA, USA). The corresponding isotype controls were analyzed in parallel: rat IgG2a, κ (PE IgG2a antibody, κ; RTK2758; BioLegend, San Diego, CA, USA), IgG1, κ (PE IgG1 antibody; RTK2071, BioLegend, San Diego, CA, USA), mouse IgG2a, κ (PE IgG2a antibody, κ; MOPC-173; BioLegend, San Diego, CA, USA) and rat IgG2b, κ (PE IgG2b, κ antibody; RTK4530; BioLegend, San Diego, CA, USA). All antibodies were spun down (10 min 50000 × *g*) before EVs staining to avoid antibody aggregates. In addition to isotype control, the following controls were used routinely for each flow cytometric measurement of EVs: 0.2 µm filtered PBS (fPBS), fPBS with mAb/isotype control, and unstained EVs (according to Welsh et al.^67^). Background noise in fPBS was minimal (Supplementary Fig. 3a). For fluorescently labeled EVs, the cytometer threshold was settled on the fluorescence channel (FL2). The flow rate during sample collecion was held at a low speed. Vesicles were analyzed using FACSDiva v8.0.1 software (BD Biosciences). To confirm the vesicular nature of detected events 1% Triton X-100 detergent (Sigma-Aldrich) was used to solubilize vesicles. Briefly, EVs from plasma were stained with CD41 PE and collected by FACSCanto with fluorescent beads (BD Cytometer Setup and Tracking Beads, BD Bioscience) detected in the FL2 channel (gate „beads”). Supplementary Fig. 3b represents the forward (FSC) and side scatter (SSC) comparison of beads (PE, 3um) with plasma EVs. Samples were collected and treated for 10 min at RT with 0.2 µm filtered Triton X100 (1%). The overlaid histogram represents intact EVs in plasma (light gray) and events remaining after TritonX-100 treatment (black, Supplementary Fig. 3c). Non-solubilized events represent less than 25% of collected events, which suggests that most of the CD41^+^ vesicles are true EVs. Number of collected fluorescent beads was comparable.

### Neutrophil and monocyte isolation from mice bone marrow

Mice were anesthetized with the mixture of ketamine and xylazine. While the mice were in deep anesthesia the cervical dislocation was performed and then femurs and tibias were isolated from which bone marrow (BM) was aseptically washed out several times with HBSS(–) (w/o Ca^2+^and Mg^2+^; Lonza Bioscience) until the BM was completely rinsed out. The procedures of neutrophil and monocyte isolation were performed as described previously^68^. Briefly, the BM cells were pelleted in a centrifuge (1300 *rpm*, 4 °C, 6 min) for hypotonic erythrocyte lysis (5 mL of ice-cold 0.2% NaCl for about 30 s, followed by 5 mL of ice-cold 1.6% NaCl to restore osmolarity) and then centrifuged at 1400 *rpm*, 4 °C for 7 min. Neutrophils and monocytes were separated by density Percoll centrifugation gradient (GE Healthcare, Arlington Heights, IL, USA). After centrifugation (2600 *rpm*, 4 °C, for 30 min) the neutrophil layer was collected between 78 and 69% and monocytes between the 52 and 69% of Percoll solution. After washing in HBSS(−) (1500 *rpm*, 4 °C, 6 min) neutrophil and monocyte pellets were resuspended in HBSS(+) (w/Ca^2+^ and Mg^2+^, Lonza Bioscience, Basel, Switzerland). The cells were stained with Trypan blue dye (Sigma–Aldrich, Saint Louis, MO, USA) to estimate their viability and counts. Purity was determined upon Türk solution staining (0.01% crystal violet in 3% acetic acid; Sigma–Aldrich, Saint Louis, MO, USA) upon cell counting in a Bürker hemocytometer. The purity of isolated neutrophils was ~ 99% and monocytes ~ 80% as estimated by their morphology (Türk/Bürker hemocytometer) and both neutrophil and monocyte viability was ~ 98% (Trypan blue). The viability and purity were determined before each ex vivo experiment. Experiments were performed in 96-well plates (Nest Scientific, Woodbridge, NJ, USA). In some experiments, coverglasses with a diameter of 5 mm (ThermoFisher Scientific, Waltham, MA, USA) were placed at the bottom of the wells of the plate, on which neutrophils were seeding at a concentration of 5 × 10^4^/well to performed subsequently immunocytochemistry. Some experiments were performed in 24-well plates (Nest Scientific, Woodbridge, NJ, USA) which were seeded with neutrophils and neutrophil co-cultures with monocytes (80% monocytes, 20% PMN) at a density of 1 × 10^6^/well. In each experimental setup, cells were first placed in an incubator (HeraeusHERAcell 150, ThermoElectron, East Brunswick, NJ, USA) with 5% CO_2_ for 30 min at 37 °C to adhere to the plates.

### Erythrocyte isolation

Erythrocytes were isolated from whole blood collected from the heart which was diluted twice with HBSS(−). The 65 and 72% Percoll solution was added with a glass Pasteur pipette and followed by diluted blood and then centrifuged (1800 *rpm*, 22 °C, 35 min). The erythrocyte pellet was suspended in HBSS(−) and centrifuged (2500 *rpm*, 22 °C, 6 min). Finally the pellet was resuspended in 1 mL HBSS(+) and red blood cells (RBCs) were counted in a Bürker hemocytometer.

### Isolation of EVs from neutrophil and erythrocyte cultures, and co-cultures of neutrophils with monocytes: ex vivo

Neutrophils or neutrophil co-cultures with monocytes were incubated for 4 h at 37 °C at a concentration of 1 × 10^6^/well. Then they were stimulated with 75 μg/mL and 10 μg/mL LPS, respectively, while erythrocytes were seeded at a concentration of 2 × 10^6^/well and stimulated with 75 μg/mL LPS in 24-well plates. After this time, the cell supernatant was collected from each culture and centrifuged for EVs isolation (400 *g* and 1200 *g*, 10 min, and then 1500 *g*, 15 min at RT). Subsequently the collected supernatant was centrifuged at 14,000 *g*, 4 °C, 35 min.

### Stimulation of neutrophils: ex vivo

Neutrophils were stimulated for 6 h (unless otherwise stated) with LPS at a final concentration of 75 μg/mL and with EVs collected from blood plasma or peritoneal lavage from healthy and endotoxemic mice (collected at 8 h of inflammation). Alternatively, EVs originating from neutrophil and erythrocyte cultures or neutrophil co-cultures with monocytes were used. Moreover, in some experiments (as indicated in the graphs), LPS was added either 30 min before/after EVs or simultaneously. In some cases, 30 min prior to EV and LPS stimulation the Siglec-E blocking antibodies (Ultra-LEAF™ purified anti-mouse Siglec-E antibody, M1305A02, BioLegend, San Diego, CA, USA) diluted 1:200 in HBSS(+) were added to the cells. EVs were used at a concentration of 50 × 10^6^ EVs/5 × 10^4^ cells/well. Subsequently, NET formation and Siglec-E expression was verified.

### Immunocytochemical staining of NETs

After stimulation with LPS and/or EVs, neutrophils placed in a 96-well plate on coverglasses were fixed in a sequence of 1%, 2% and 3% paraformaldehyde (PFA; Alfa Aesar, Haverhill, MA, USA) in PBS for 2, 10 and 20 min, respectively, and then washed with PBS for 5 minutes^69^. The coverglasses were subsequently incubated in a blocking solution, namely 3% bovine serum albumin (BSA; Sigma–Aldrich, Saint Louis, MO, USA) in PBS for 45 min at RT in a humid chamber. Next, NETs components were labelled with rabbit polyclonal anti-histone H3 (citrulline R2 + R8 + R17) (Abcam, Cambridge, UK) or anti-neutrophil elastase (Abcam, Cambridge, UK) antibodies diluted 1:200 in 1% BSA/PBS and incubated overnight at 4 °C in a humid chamber. The coverglasses were then washed in PBS and incubated with secondary Cy3-conjugated goat anti-rabbit IgG (H + L) (Jackson Immunoresearch Laboratories, Ely, UK) diluted 1:300 in 1% BSA/PBS for 1 h at RT in the dark. After this time, the fluorescent dye Sytox green (5 µM; Invitrogen, Carlsbad, CA, USA) was added to visualize extracellular DNA. After 5 min of staining, the coverglasses were washed in PBS and mounted with VECTASHIELD Mounting Medium (Vector Laboratories, Burlingame, CA, USA). Fluorescent signal was detected with a ZEISS Axio Examiner.Z1 equipped with DSD2 (Andor, Oxford Instruments, Abingdon, UK). Obtained images were converted to grayscale—16-bit type using ImageJ software. The positive signal (black on white) from exDNA/NET components was then estimated and expressed as percentage of FOV covered area as described above.

### Siglec-E immunostaining

Prior to staining, neutrophils seeded on coverglasses were fixed as described above. Nonspecific antibody binding was blocked by incubation with 3% BSA/PBS for 45 min in RT. Subsequently, the cells were labeled with recombinant rat monoclonal antibody anti-Siglec-E (PE, M1304A01; BioLegend, San Diego, CA, USA) diluted 1:50 in 1% BSA/PBS and incubated overnight at 4 °C in a humid chamber. The slides were then washed 2 times in PBS and were mounted with VECTASHIELD Mounting Medium. Fluorescent signal was detected with a ZEISS Axio Examiner.Z1 equipped with DSD2 (Andor, Oxford Instruments, Abingdon, UK). Obtained images were converted to grayscale—16-bit type using ImageJ software. The positive signal (black on white) from exDNA/NET components was then estimated and expressed as percentage of FOV covered area as described above.

### Statistical analyses

All data are presented as mean values ± SD. Shapiro–Wilk test was performed to determine the normality of the data. Data were compared either by using the parametric unpaired two tailed Student’s *t* test or one-way analysis of variance (ANOVA) with Bonferroni multiple comparisons post hoc test. In the case of data not normally distributed the nonparametric Mann–Whitney U test, or the Kruskal–Wallis test followed by Dunn’s test were used. Statistical significance was set at *p* < 0.05. The number of mice or repetitions is indicated in each figure.

### Supplementary Information

Supplementary Information 1.

Supplementary Video S1.

Supplementary Video S2.

Supplementary Video S3.

Supplementary Video S4.

Supplementary Video S5.

Supplementary Video S6.

## Data Availability

All data is contained within the manuscript and the supplement.
