# Supplementary material for: Large extracellular vesicle (EV) and neutrophil extracellular trap (NET) interaction captured in vivo during systemic inflammation
Source: Sci Rep. 2024 Feb 26;14:4680. doi: 10.1038/s41598-024-55081-x (PMC10897202; doi:10.1038/s41598-024-55081-x)
Supplement: Supplementary file 1 — Supplementary Information 1. [file 41598_2024_55081_MOESM1_ESM.pdf]

Supplementary Figure S1

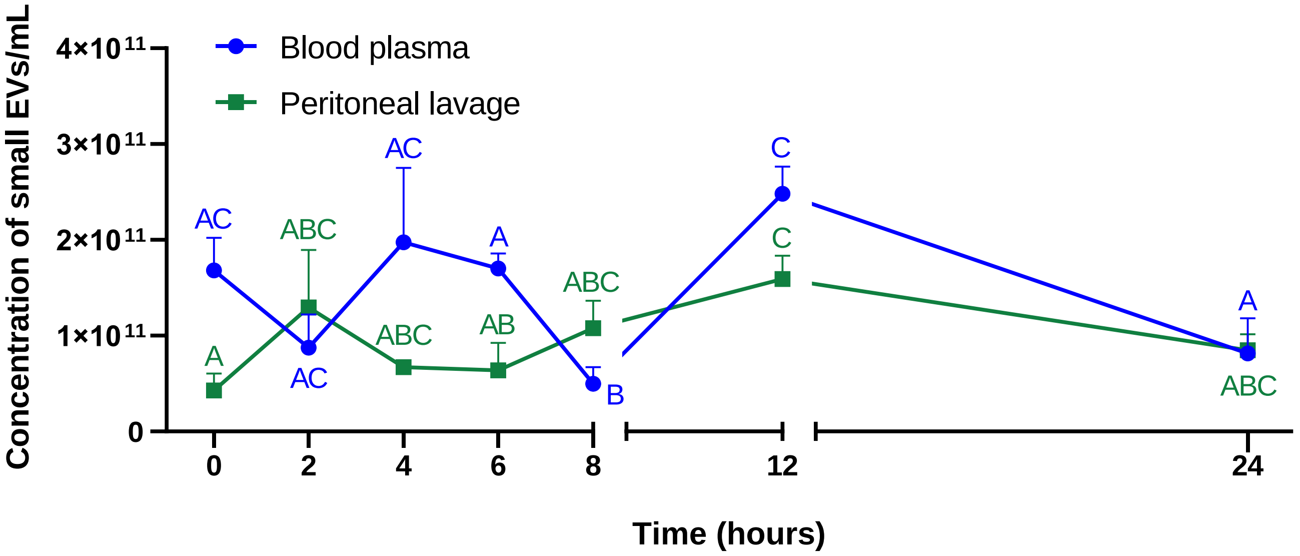

Supplementary Figure S1 Quantification of the concentration of smaller extracellular vesicles (small EVs) during homeostasis (0 hrs) and lipopolysaccharide (LPS)-induced systemic inflammation (*i.p.* 1 mg/kg b.w.) in C57BL/6J mice. Concentration of small EVs was estimated *ex vivo* with nanoparticle tracking analysis (NTA) in samples collected from blood plasma and peritoneal lavage. The results are expressed as the mean values ± SD. Values significantly different ($p<0.05$) according to one-way ANOVA with Bonferroni multiple comparisons *post hoc* test are designated by letters (different letters indicate statistical differences); n=3.

Supplementary Figure S2

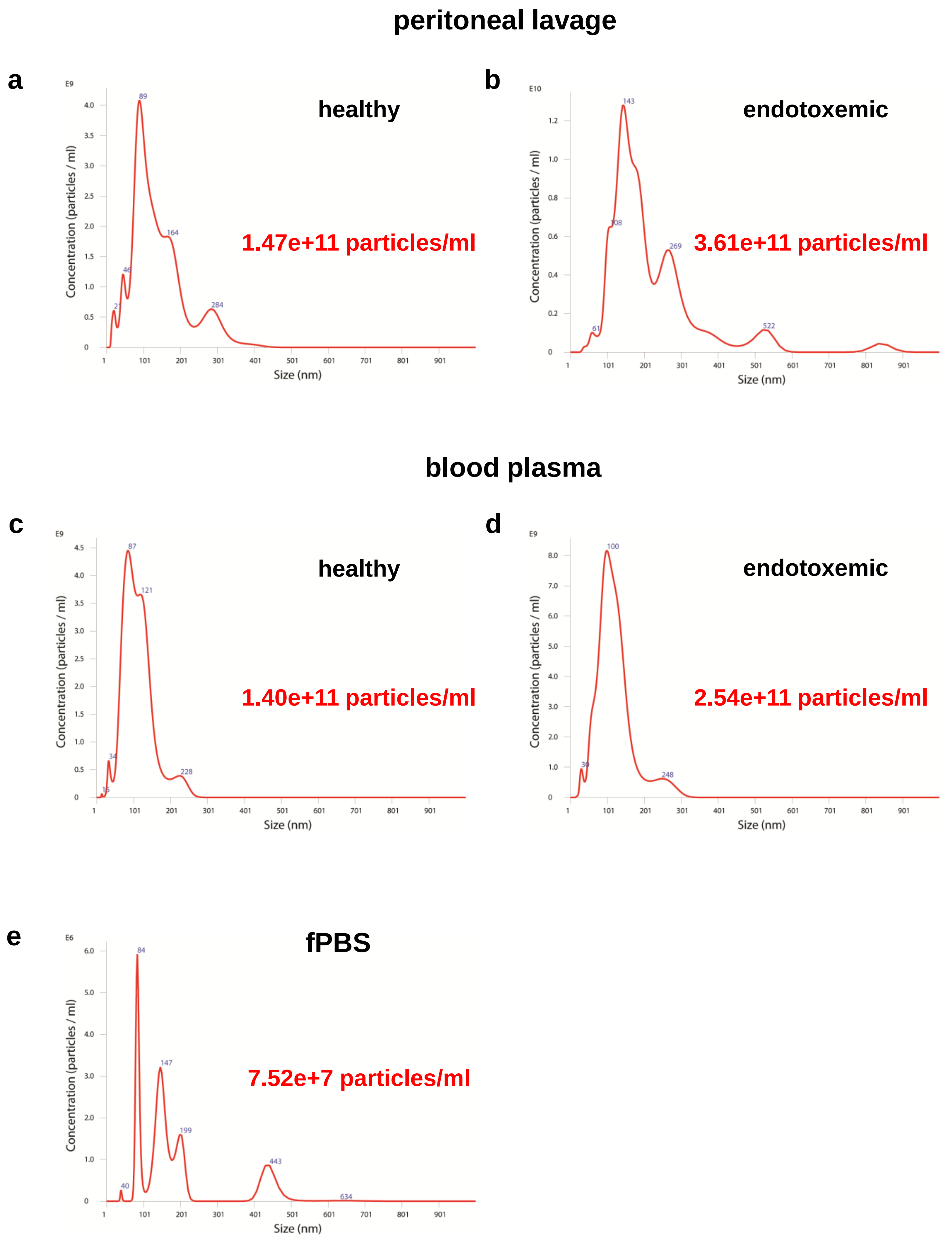

Supplementary Figure S2 Characteristics of extracellular vesicles (EVs) by nanoparticle tracking analysis (NTA). Representative NTA histograms of the size distribution of EVs derived from (**a**,**c**) healthy (0 hrs) and (**b**,**d**) endotoxemic (8 hrs) C57BL/6J mice. EVs were isolated from (**a**,**b**) peritoneal lavage and (**c**,**d**) blood plasma. (**e**) Paricles present in 0.2 μm filtered PBS (fPBS).

Supplementary Figure S3

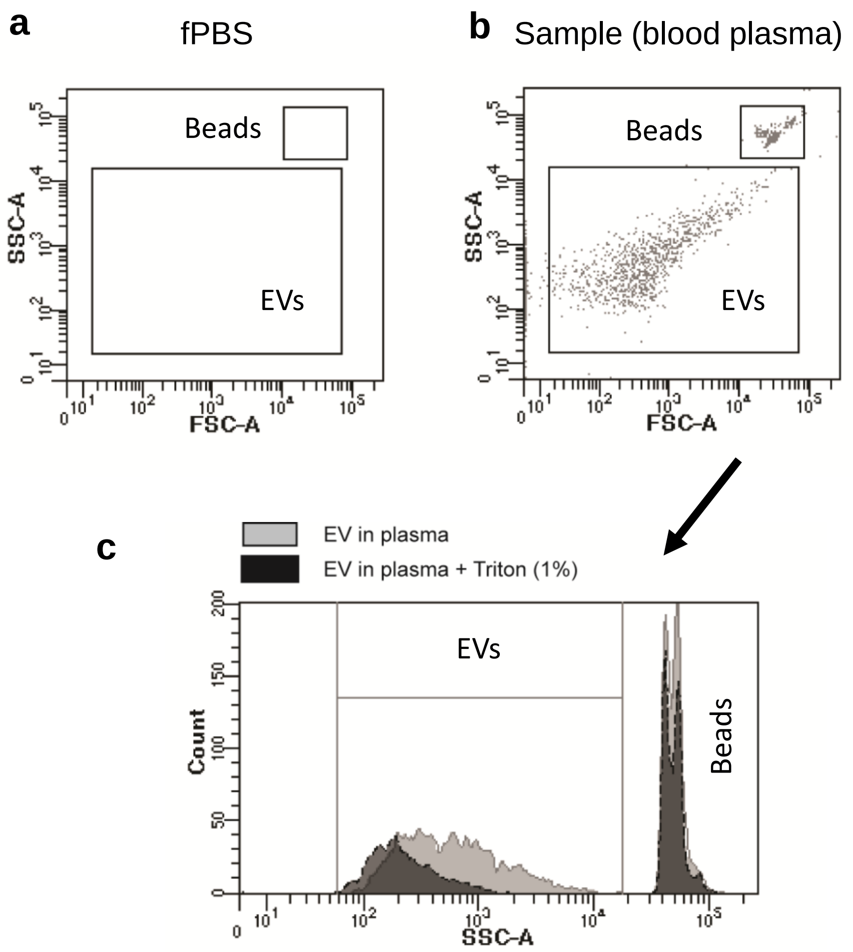

Supplementary Figure S3 Control studies for extracellular vesicle (EV) characterization with flow cytometry. (**a**-**b**) Representative forward (FSC) and side scatter (SSC) comparison of filtered PBS (fPBS), beads (PE, 3um) and blood plasma EVs. (**a**) Minimal background noise in 0.2 µm fPBS *versus* (**b**) control beads (the insert) and exemplary sample (blood plasma). (**c**) To determine the vesical structure of plasma EVs, samples were treated with 1% Triton X-100. The overlaid histogram represents intact EVs in plasma (light gray) and EVs after Triton X-100 treatment (black). The read outs were performed with FACSCanto flow cytometer.

Supplementary Figure S4

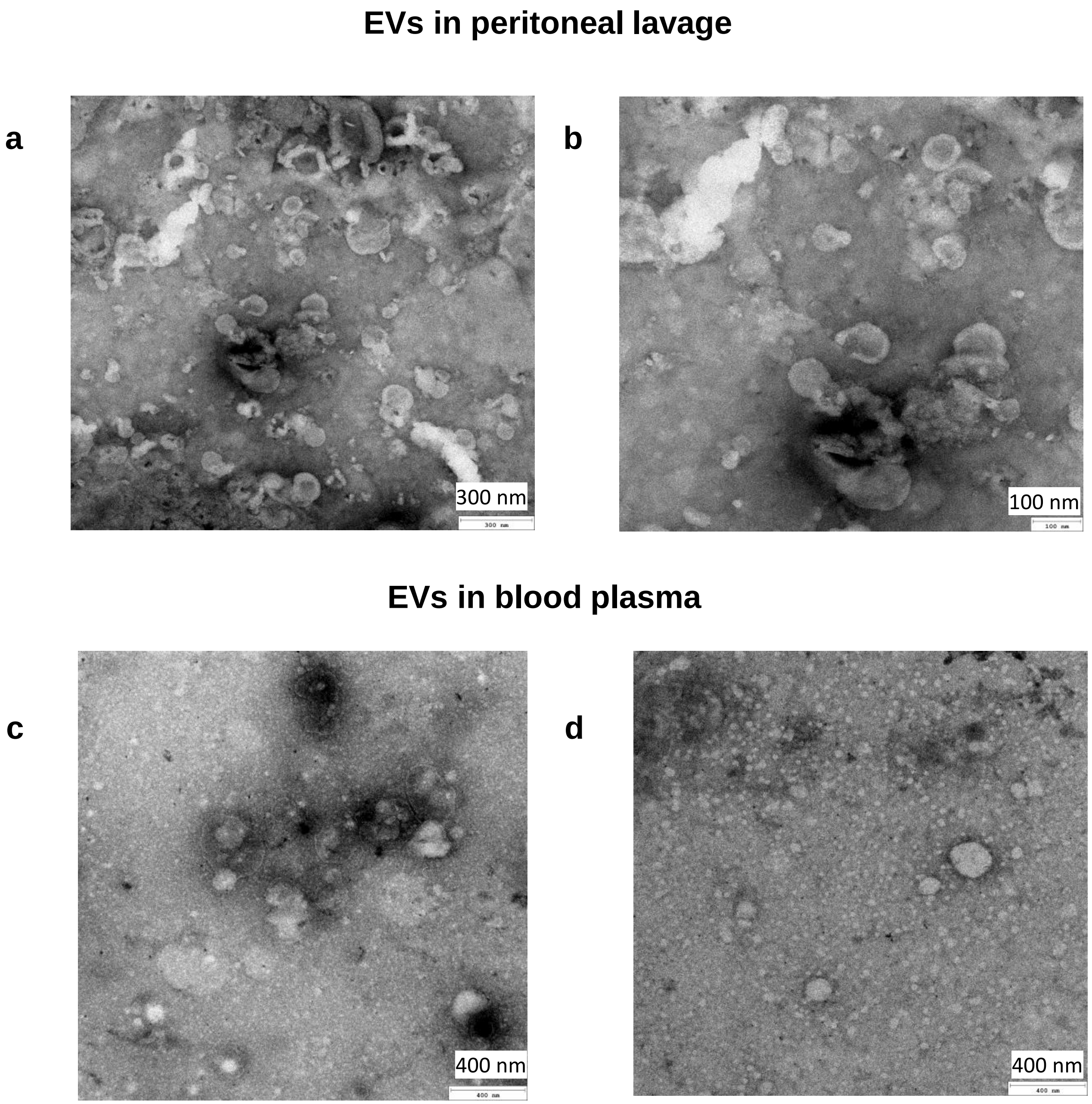

Supplementary Figure S4 Characterization of extracellular vesicles (EVs) by transmission electron microscopy (TEM). Representative images of EVs isolated from endotoxemic (8 hrs) C57BL/6J mice from either (**a**) peritoneal lavage or (**b**) blood plasma.

Supplementary Figure S5

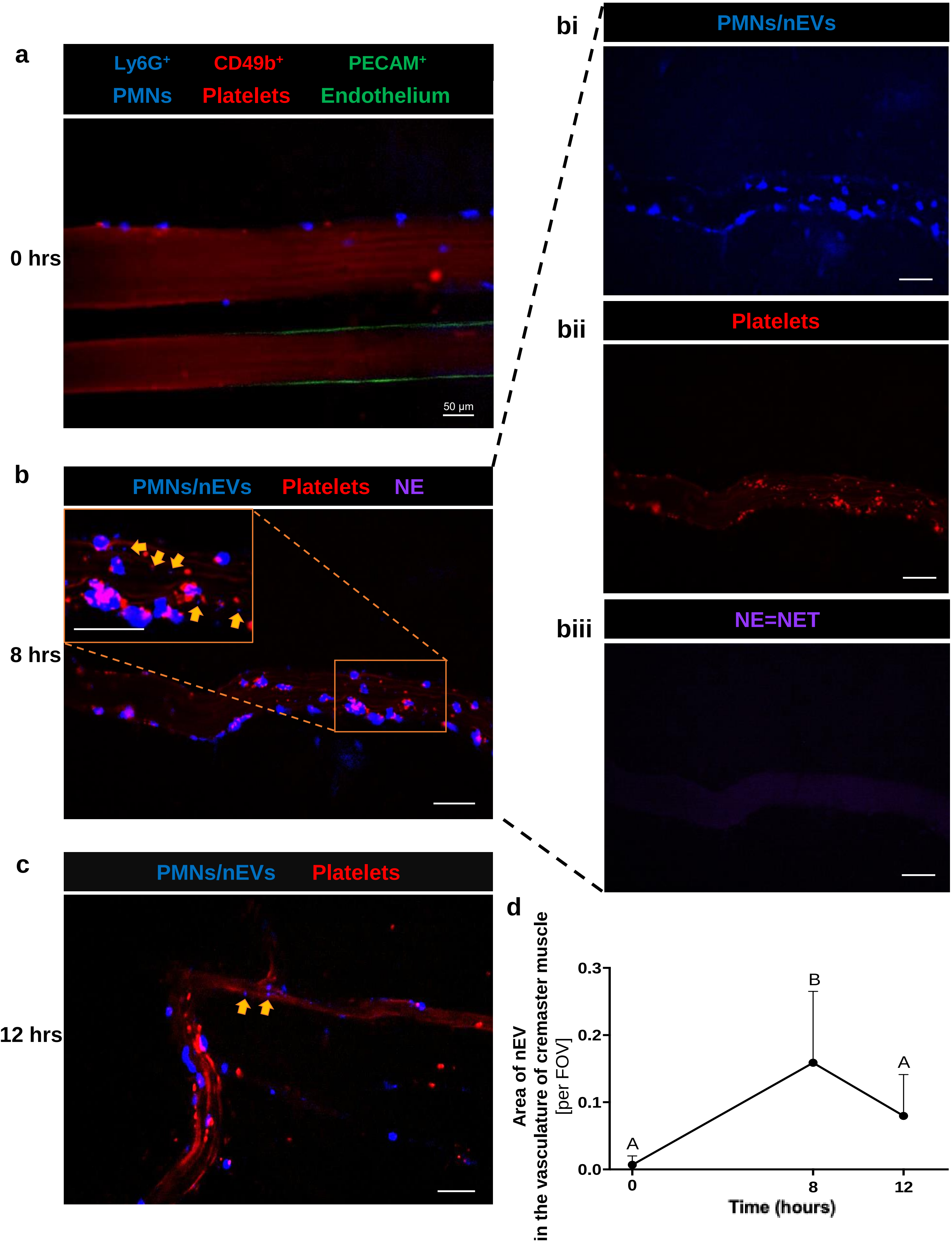

Supplementary Figure S5 Presence/deposition of neutrophils/neutrophil-derived extracellular vesicles (nEVs) and neutrophil extracellular traps (NETs) in the vasculature of cremaster muscle of healthy and endotoxemic C57BL/6J mice. Representative images of EVs and NETs were acquired with Spinning Disk Confocal Intravital Microscopy (SD-IVM) during (**a**) homeostasis (neutrophils, PMNs – blue; platelets – red; $PECAM^{+}$ endothelial cell layer – green), (**b**) 8 and (**c**) 12 hrs of lipopolysaccharide (LPS)-induced endotoxemia (*i.p.* 1 mg/kg b.w.). Selected area of the image presented in panel **b** was magnified to visualize all cells/structures (exemplary EVs are marked with yellow arrows). Single channels of the above image depict (**bi**) nEVs and neutrophils (blue), (**bii**) platelets (red), and (**biii**) lack of neutrophil elastase (otherwise – violet signal). The scale bar indicates 50 µm. (**d**) Quantification of nEV area (%) achieved with ImageJ v1.53a software. The results are expressed as the mean values ± SD. Values significantly different ($p<0.05$) according to Kruskal-Wallis's test followed by Dunn's test are designated by letters (different letters indicate statistical differences); n=3. FOV – field of view.

Supplementary Figure S6

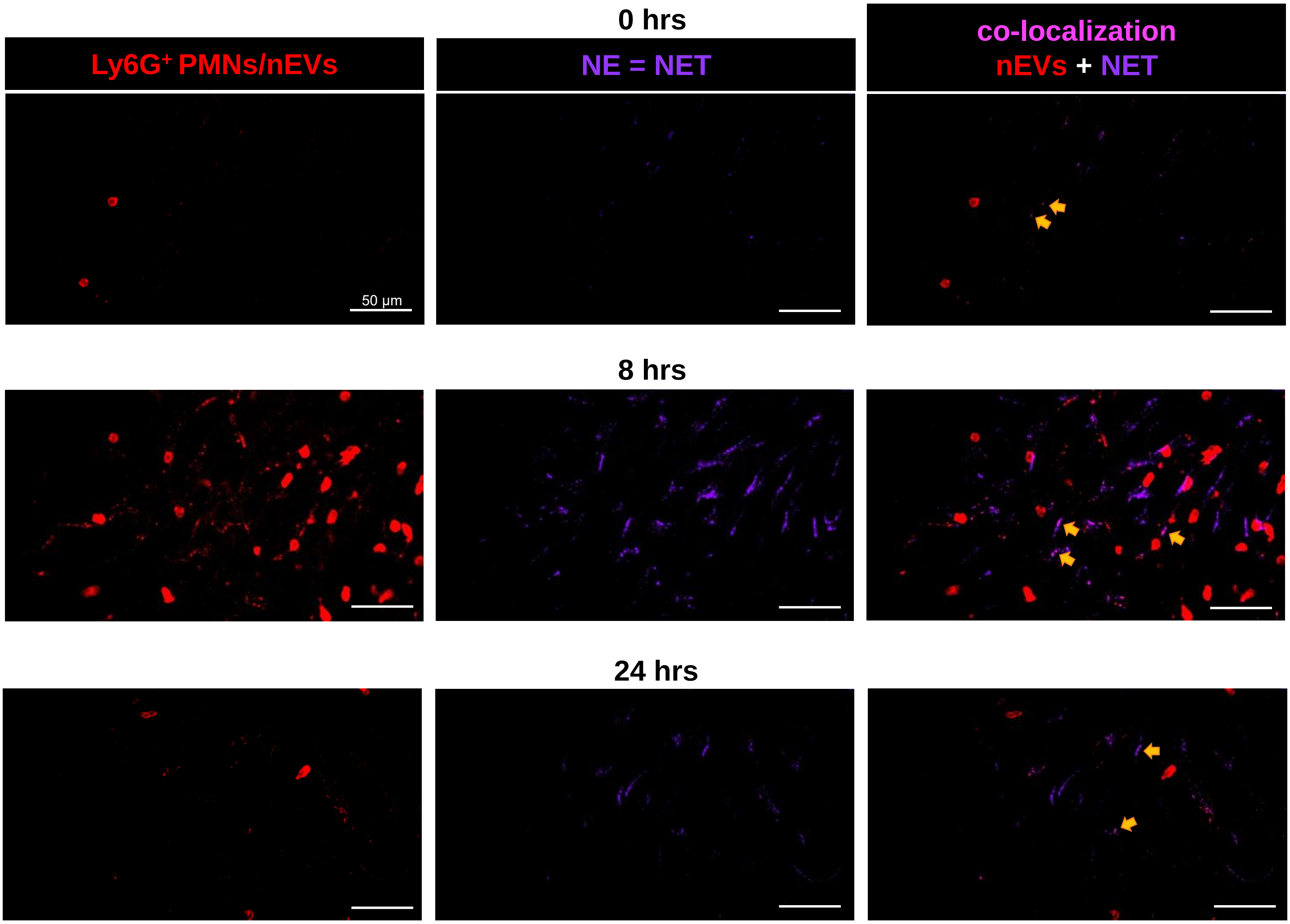

Supplementary Figure S6 Presence/deposition of neutrophils/neutrophil-derived extracellular vesicles (nEVs) and neutrophil extracellular traps (NETs) in liver sinusoids of healthy and lipopolysaccharide (LPS)-induced systemic inflammation (*i.p.* 1 mg/kg b.w.) in C57BL/6J mice. Representative images of EVs and NETs were imaged with Spinning Disk Confocal Intravital Microscopy (SD-IVM) during homeostasis and at specific time points of endotoxemia. Representative images revealing differences in nEVs, NET deposition (NE) and their co-localization as well as neutrophil numbers in liver sinusoids. Single channels depicting nEVs (red, small dots) and neutrophils themselves (PMN; red, large cells) – left panel, neutrophil elastase = NET (violet granules) – middle panel and EV+NET co-localization (pink; exemplary co-localization are marked with yellow arrows) – right panel. The photos were taken under optical magnification of 40x. The scale bar indicates 50 µm.

Supplementary Figure S7

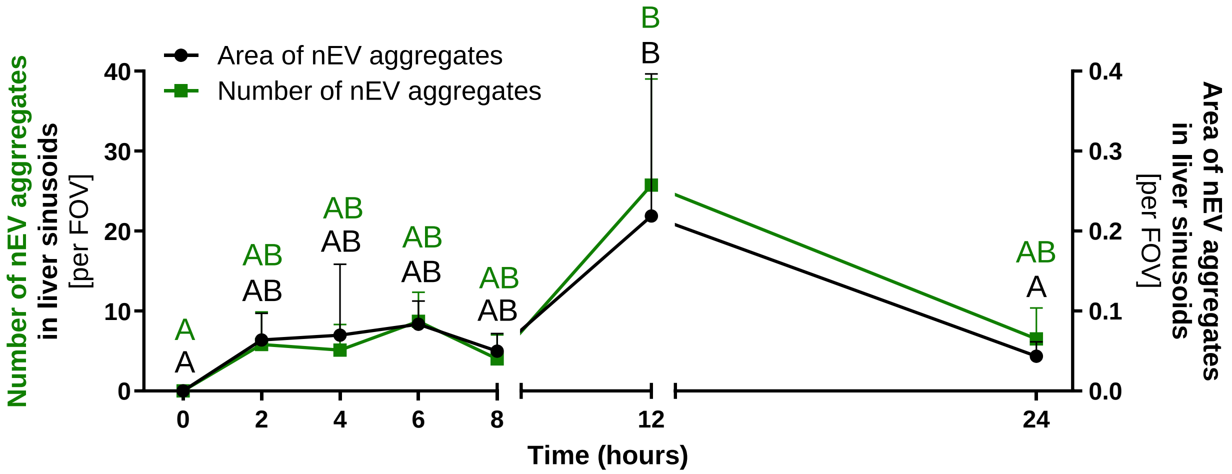

Supplementary Figure S7 Quantification of the number/area (%) of neutrophil extracellular vesicle aggregates (nEV aggregates) during homeostasis and lipopolysaccharide (LPS)-induced systemic inflammation (*i.p.* 1 mg/kg b.w.) in C57BL/6J mice. Number/area (%) of nEV aggregates were *in vivo* acquired with Spinning Disk Confocal Intravital Microscopy (SD-IVM) directly in vasculature (sinusoids) of mice liver and analyzed with ImageJ v1.53a software. The results are expressed as the mean values ± SD. Values significantly different ($p<0.05$) according to Kruskal-Wallis's test followed by Dunn's test are designated by letters (different letters indicate statistical differences); n=3. FOV – field of view.

Supplementary Figure S8

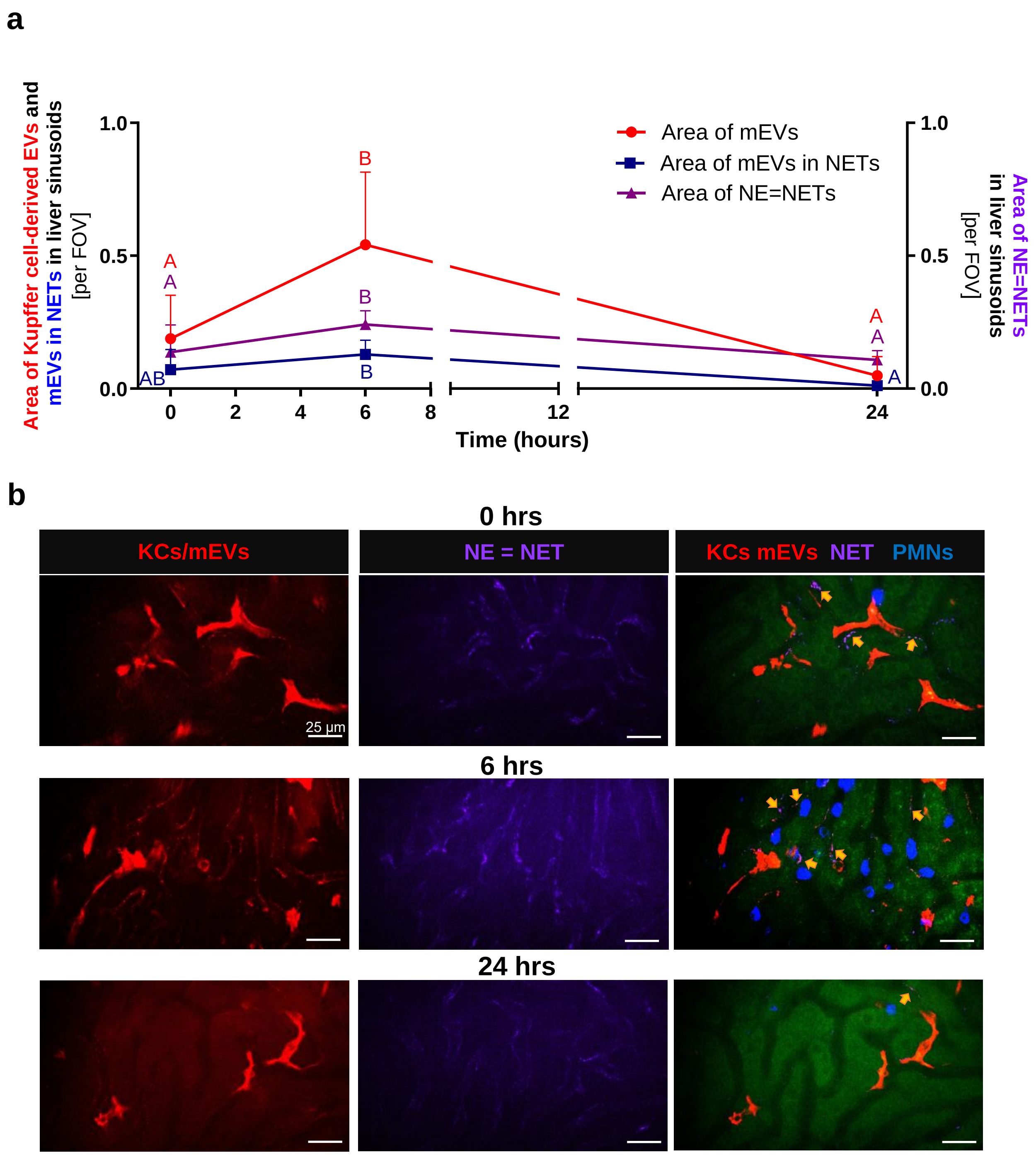

Supplementary Figure S8 Presence/deposition of monocyte/macrophage extracellular vesicles (mEVs) and neutrophil extracellular traps (NETs) during homeostasis and lipopolysaccharide (LPS)-induced systemic inflammation (*i.p.* 1 mg/kg b.w.) in C57BL/6J mice. (**a**) Quantification of the changing area (%) covered by mEV (red line); neutrophil elastase (NE) = NET (violet line) and EVs present in NETs – co-localization (blue line) was calculated by obtaining *in vivo* images directly in sinusoids of liver and analyzed with ImageJ v1.53a software. The results are expressed as the mean values ± SD. Values significantly different (*p<0.05*) according to Kruskal-Wallis's test followed by Dunn's test are designated by letters (different letters indicate statistical differences); n=3. FOV – field of view. (**b**) Representative images were acquired with Spinning Disk Confocal Intravital Microscopy (SD-IVM) showing mEV (red, small dots) and Kupffer cells (KC; red cells) – left panel, NE = NET (violet granules) – middle panel, and co-localization of EVs and NET component (pink; marked with yellow arrows) as well as neutrophils (PMN; blue cells) within sinusoids of liver, the frames from each channel were overlaid – right panel. On images, autofluorescent hepatocytes (dim green) can be observed in between which sinusoids are localized (black ducts). The photos were taken under optical magnification of 63x. The scale bar indicates 25 μm.

Supplementary Figure S9

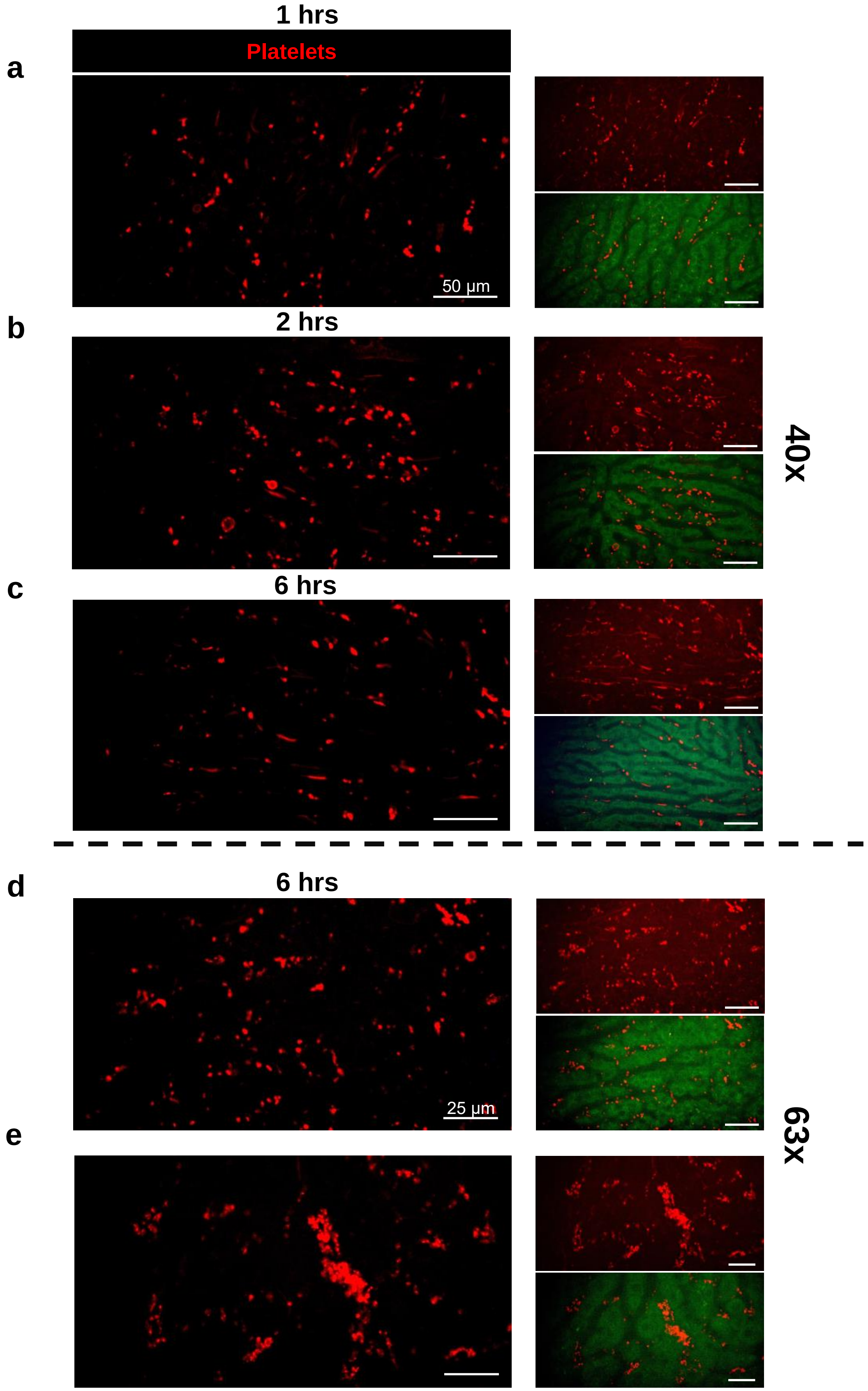

Supplementary Figure S9 Representative images of platelets in mouse liver sinusoids. C57BL/6J mice with lipopolysaccharide (LPS)-induced endotoxemia (*i.p.* 1 mg/kg b.w.) were subjected to *in vivo* imaging with Spinning Disk Confocal Intravital Microscopy (SD-IVM) at individual time points (1, 2, 6 hrs). Single channels represent platelets and no platelet-derived EVs (pEVs) imaged at optical magnification (**a**-**c**) 40x and (**d**,**e**) 63x. The right panel shows downgrade images of the left panel: top photos without and bottom photos with background channel correction. On images, autofluorescent hepatocytes (dim green) can be observed in between which sinusoids are localized (black ducts).

Supplementary Figure S10

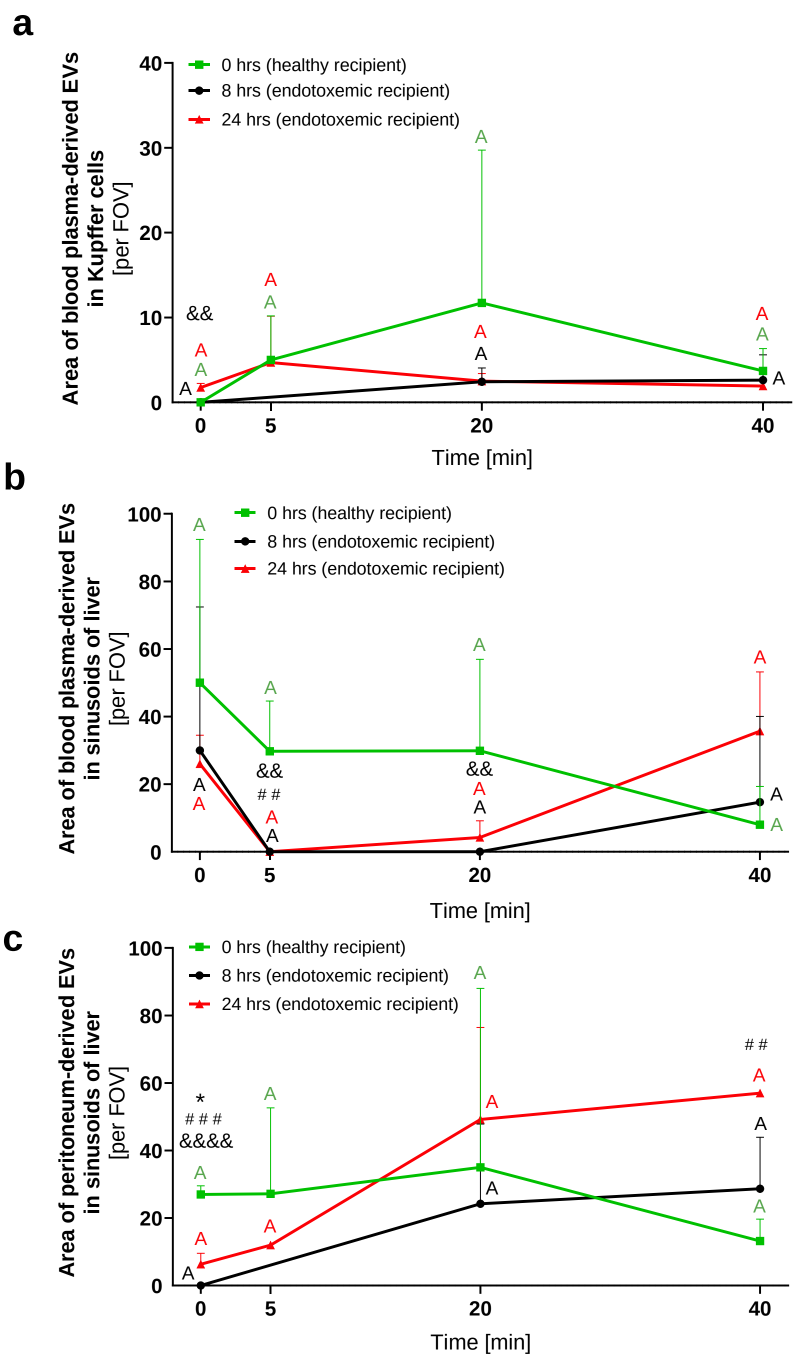

Supplementary Figure S10 Kinetics of the surface covered by exogenously labeled extracellular vesicles (EVs) in mice liver. The exogenously labeled EVs from blood plasma and peritoneal lavage of C57BL/6J mice with lipopolysaccharide (LPS)-induced endotoxemia (*i.p.*1 mg/kg b.w.; 8 hrs post LPS inoculation) in liver of healthy mice (0 hrs) and mice with 8- and 24-hours endotoxemia were subjected to *in vivo* imaging with Spinning Disk Confocal Intravital Microscopy (SD-IVM). EVs derived from (**a**) blood plasma in hepatic macrophages (Kupffer cells) and EVs derived from (**b**) blood plasma and (**c**) peritoneal lavage in liver sinusoids of healthy (green line) and endotoxemic (8 hrs – black line and 24 hrs – red line) mice. Intravenously administered, exogenously labeled EVs were observed for 40 minutes after administration (0 min). The EV area was estimated at selected time points (0, 5, 20 and 40 min) after injection of exogenous labelled EVs. Values significantly different (*p<0.05*) according to one-way ANOVA with Bonferroni multiple comparisons *post hoc* test are designated by letters (different letters indicate statistical differences). Asterisks and symbols equivalent to asterisks, such as: #, & indicates statistically significant differences (between the study groups at particular time points, i.e., & 0 hrs vs. 8 hrs; # 0 hrs vs. 24 hrs; * 8 hrs vs. 24 hrs) according to unpaired two-tailed Student's t-test (** *p≤0.01*, *** *p≤0.001*, **** *p≤0.0001*); n=3. FOV – field of view.

Supplementary Figure S11

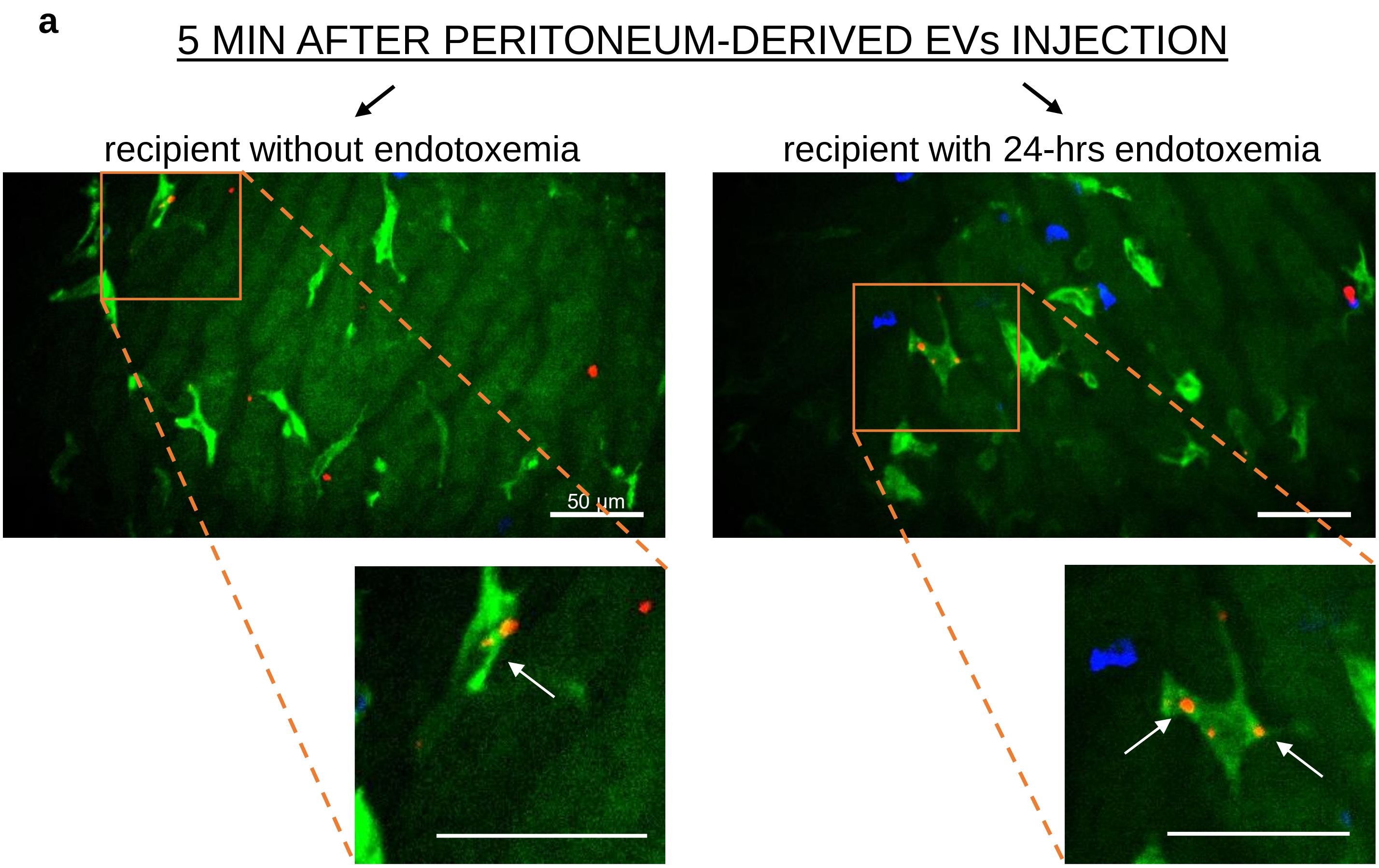

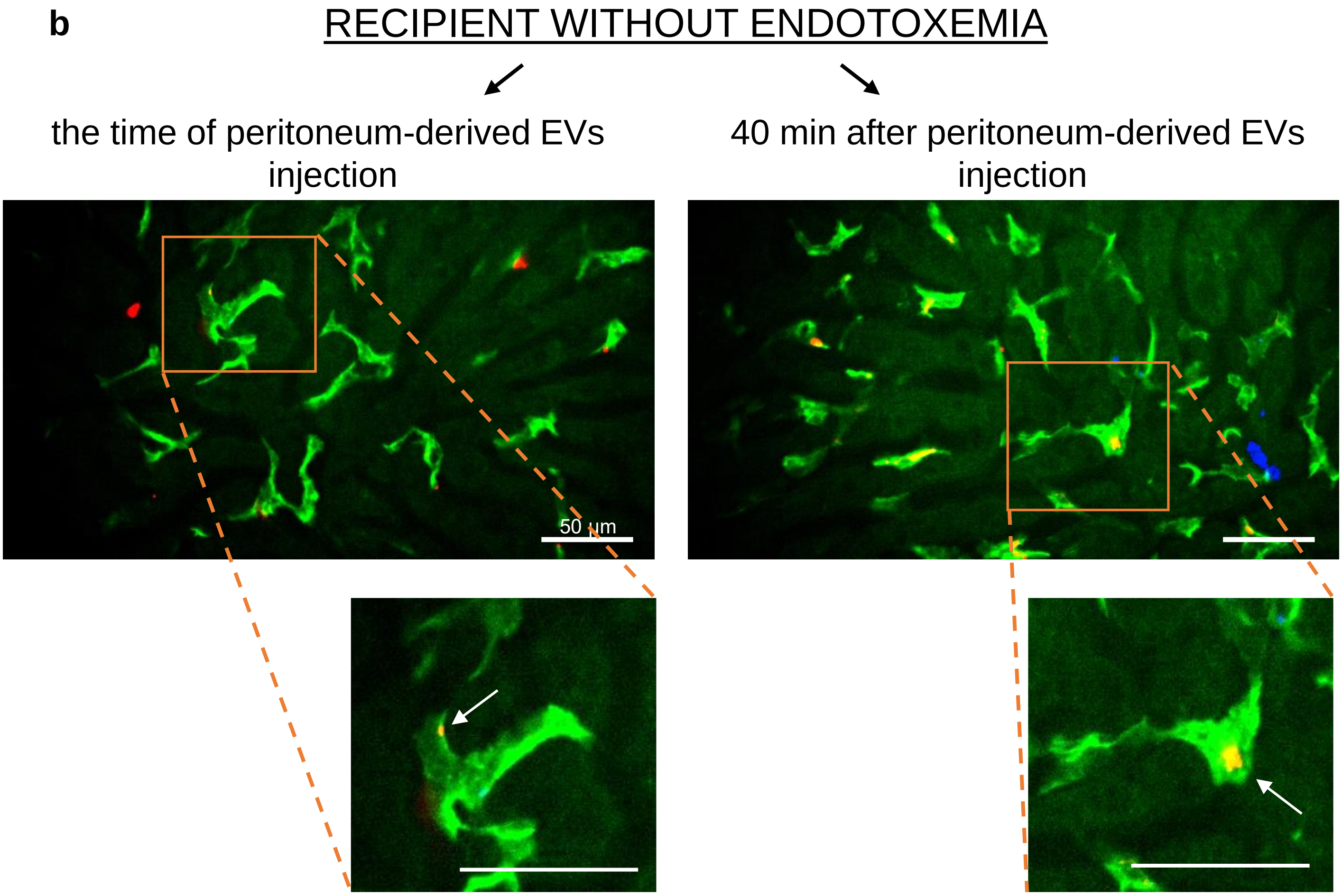

Supplementary Figure S11 Representative images of exogenously labeled extracellular vesicles (EVs) in hepatic macrophages (Kupffer cells). Peritoneal lavage-derived EVs (red structures) in (**a**) hepatic macrophages (light green cells against a background of dim green autofluorescent liver) 5 minutes after intravenous administration to healthy and endotoxemic (24 hrs) recipients, and in (**b**) a healthy recipient during intravenous administration (0 min) and after 40 min of imaging. EVs present in Kupffer cells are marked with white arrows. The photos were taken under optical magnification of 40x. The scale bar indicates 50 μm.

Supplementary Figure S12

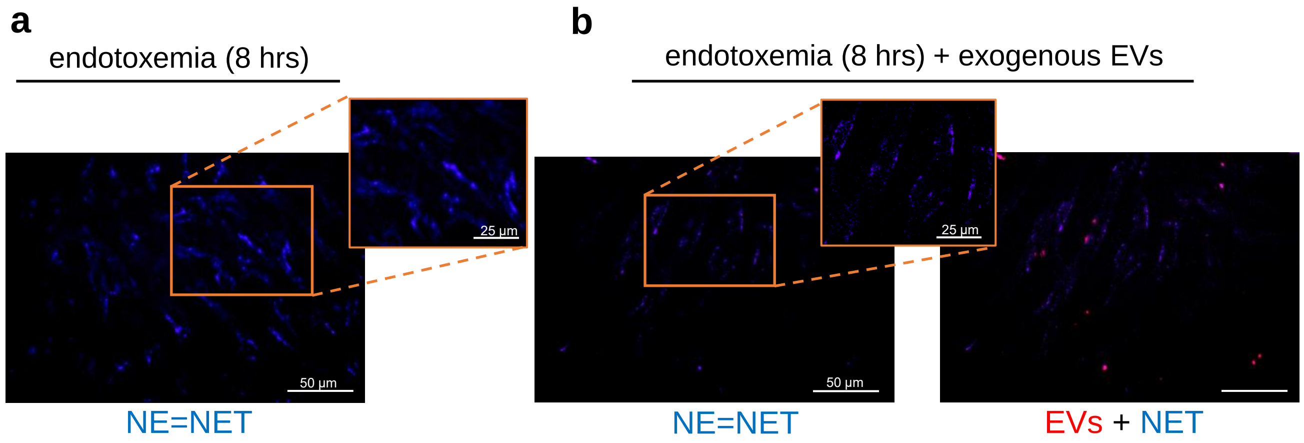

Supplementary Figure S12 Presence/deposition of neutrophil extracellular traps (NETs) and exogenously labeled extracellular vesicles (EVs) in liver sinusoids. C57BL/6J healthy mice with 8-hour lipopolysaccharide (LPS)-induced endotoxemia (*i.p.* 1 mg/kg b.w.) were subjected to *in vivo* imaging with Spinning Disk Confocal Intravital Microscopy (SD-IVM). Representative images of NET (blue) deposition obtained from mice that (**a**) were only treated with LPS (8 hrs) and those that (**b**) additionally received intravenously labeled exogenous EVs (red) derived from peritoneal lavage. The images were taken under optical magnification of 40x. The scale bar indicates 25 and 50 µm.

Supplementary Figure S13

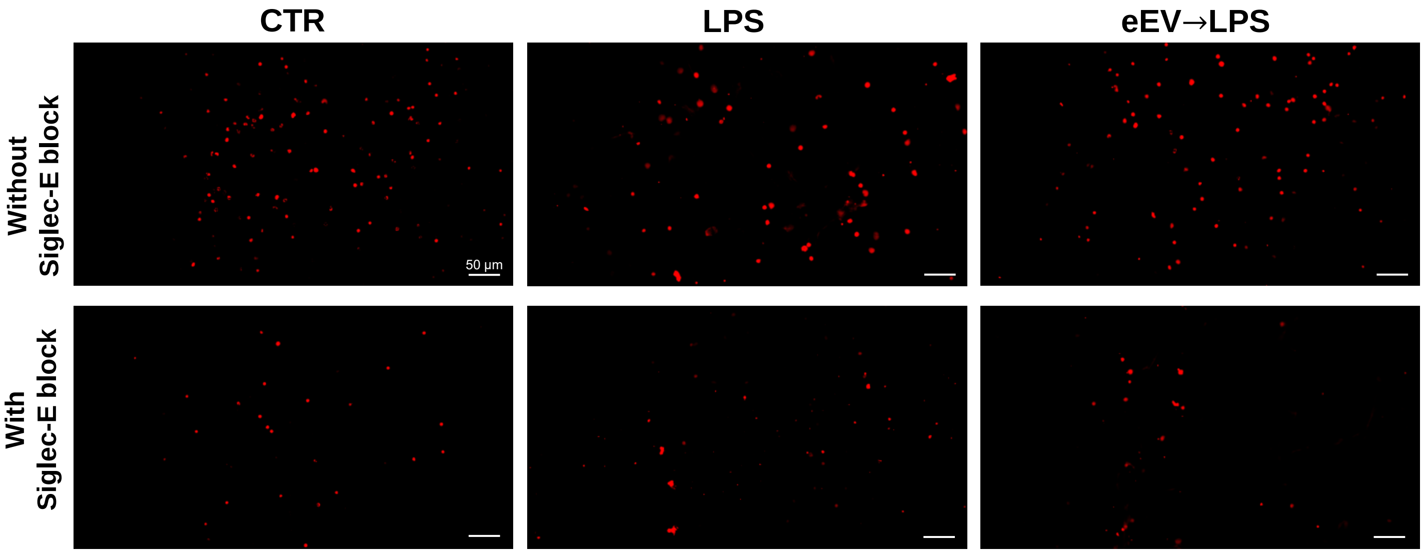

Supplementary Figure S13 Representative images of Siglec-E signal on mouse neutrophils in response to erythrocyte extracellular vesicles (eEVs). EVs were collected from previously isolated erythrocytes obtained from blood of healthy (0 hrs) C57BL/6J mice. The isolated cells were stimulated *ex vivo* with lipopolysaccharide (LPS; 75 µg/mL; 4-hour incubation) to induce the secretion of EVs. The obtained EVs (cell supernatant) were added to the neutrophils isolated from the bone marrow (6-hour incubation). The following groups were tested: unstimulated cells (CTR); LPS-stimulated cells (75 µg/mL); eEVs at a density of 50×10^6 EVs/10^5 cells (30 min) before adding LPS (eEVs→LPS). Prior (30 min) to EVs and LPS stimulation neutrophils were incubated without (upper panel) or with (lower panel) the Siglec-E blocker. Siglec-E (red) were visualized by PE anti-Siglec-E antibody (diluted 1:50). The photos were taken under optical magnification of 20x. The scale bar indicates 50 µm.

Supplementary Video S1
EVs in the mouse vasculature of cremaster muscle during homeostasis and endotoxemia. Mice were injected intraperitoneally (*i.p.*) with LPS (1 mg/kg b.w.) and their cremaster muscle was subjected to intravital imaging. Prior to imaging antibodies staining Ly6G neutrophil (PMN) and neutrophil-derived EVs (Brilliant Violet 421, blue; exemplary EVs indicated by yellow arrows), CD49b platelets (PE, red) and CD31 (PECAM) on endothelial cells (Alexa Fluor 488, green) were injected via the jugular vein. Each video was recorded at 20x optical magnification for approximately 10-15 minutes. Scale 50 µm.

Supplementary Video S2
Moving and rolling of EVs along endothelium in the vasculature of cremaster muscle during endotoxemia. Mice were injected intraperitoneally (*i.p.*) with LPS (1 mg/kg b.w.; 8 hrs) and their cremaster muscle was subjected to intravital imaging. Prior to imaging antibodies staining Ly6G neutrophil (PMN) and neutrophil-derived EVs (PE, red; exemplary EVs indicated by yellow arrows) were injected via the jugular vein. Each video was recorded at 20x optical magnification for approximately 10-15 minutes. Enlarged video fragments have been slowed down 4 times. Scale 50 µm.

Supplementary Video S3
Entrapped EVs in ejected NETs in liver sinusoids of endotoxemic mice. Mice were injected intraperitoneally (*i.p.*) with LPS (1 mg/kg b.w.) and their livers were subjected to intravital imaging. Prior to imaging antibodies staining Ly6G+ neutrophils (PMN) and neutrophil-derived EVs (PE, red), and neutrophil elastase (NE)=NETs (Alexa Fluor 647, violet) were injected via the jugular vein. Exemplary co-localization of EVs in NETs (pink) are marked with yellow arrows. Superimposed channels of recorded videos with the autofluorescent hepatocyte channel turned on (video numbers 1-5(a)) and turned off (video numbers 1-5(b)). Videos numbered 1-3(c) show enlarged fragments of videos 1-3(b), respectively. Video 6 shows EVs anchoring just after being secreted into NETs present in blood vessels; 6a single channel representing PMN/EVs, 6b – superimposed channels from PMNs/EVs and NE=NET. Videos were recorded at 20x (video numbers 1-3) and 40x (video numbers 4 -6) optical magnification for approximately 10-15 minutes. Scale 25 µm (video numbers 4-6) and 50 µm (videos number 1-3) .

Supplementary Video S4
Release of EVs in real time in liver sinusoids during homeostasis and endotoxemia. Mice were injected intraperitoneally (*i.p.*) with LPS (1 mg/kg b.w.) and their livers were subjected to intravital imaging. Prior to imaging antibodies staining Ly6G neutrophil (PMN) and neutrophil-derived EVs (PE, red; exemplary EVs indicated by yellow arrows) were injected via the jugular vein. Each video was recorded at 20x optical magnification for approximately 10-15 minutes. Enlarged video fragments have been slowed down 4 times. Scale 50 µm.

Supplementary Video S5
Moving and rolling of EVs along endothelium in the mouse liver during endotoxemia. Mice were injected intraperitoneally (*i.p.*) with LPS (1 mg/kg b.w.) and their livers were subjected to intravital imaging. Prior to imaging antibodies staining Ly6G neutrophil (PMN) and neutrophil-derived EVs (PE, red; exemplary EVs indicated by yellow arrows) were injected via the jugular vein. Each video was recorded at 20x optical magnification for approximately 10-15 minutes. Some fragments were enlarged. Scale 50 µm.

Supplementary Video S6
(I) EV and cellular interactions and (II) phagocyted EVs by Kupffer cells in liver sinusoids during endotoxemia. Mice were injected intraperitoneally (*i.p.*) with LPS (1 mg/kg b.w.; 6 hrs) and their livers were subjected to intravital imaging. Prior to imaging antibodies staining Ly6G neutrophil (PMN) and neutrophil-derived EVs (Alexa Fluor 488, green), F4/80 Kupffer cells (eFluor 660, violet) and CD49b platelets (PE, red) were injected via the jugular vein. Exemplary interactions and engulfed EVs (blue) are indicated by yellow arrows. Each video was recorded at 63x optical magnification for approximately 10-15 minutes. Scale 50 µm.
